# Supplementary material for: Understanding the implementation of ‘sick day guidance’ to prevent acute kidney injury across a primary care setting in England: a qualitative evaluation
Source: BMJ Open. 2017 Nov 8;7(11):e017241. doi: 10.1136/bmjopen-2017-017241 (PMC5695520; doi:10.1136/bmjopen-2017-017241)
Supplement: Author's manuscript [file bmjopen-2017-017241.draft_revisions.pdf]

BMJ Open is committed to open peer review. As part of this commitment we make the peer review history of every article we publish publicly available.

When an article is published we post the peer reviewers' comments and the authors' responses online. We also post the versions of the paper that were used during peer review. These are the versions that the peer review comments apply to.

The versions of the paper that follow are the versions that were submitted during the peer review process. They are not the versions of record or the final published versions. They should not be cited or distributed as the published version of this manuscript.

BMJ Open is an open access journal and the full, final, typeset and author-corrected version of record of the manuscript is available on our site with no access controls, subscription charges or pay-per-view fees (http://bmjopen.bmj.com).

If you have any questions on BMJ Open's open peer review process please email editorial.bmjopen@bmj.com

BMJ Open

Implementation of 'sick day guidance' to prevent community-based Acute Kidney Injury: a qualitative study

Journal:	*BMJ Open*
Manuscript ID	bmjopen-2017-017241
Article Type:	Research
Date Submitted by the Author:	18-Apr-2017

Complete List of Authors:	Martindale, Anne-Marie; National Institute for Health Research Collaboration for Leadership in Applied Health Research and Care Greater Manchester; The University of Manchester<br>Elvey, Rebecca ; National Institute for Health Research Collaboration for Leadership in Applied Health Research and Care Greater Manchester; The University of Manchester<br>Howard, Susan; National Institute for Health Research Collaboration for Leadership in Applied Health Research and Care Greater Manchester; Salford Royal NHS Foundation Trust<br>McCorkindale, Sheila; NHS Salford Clinical Commissioning Group<br>Sinha, Smeeta; Salford Royal NHS Foundation Trust, Stott Lane, M6 8HD<br>Blakeman, Tom; National Institute for Health Research Collaboration for Leadership in Applied Health Research and Care Greater Manchester; The University of Manchester

**Primary Subject Heading**:	General practice / Family practice
Secondary Subject Heading:	Communication, Qualitative research
Keywords:	Acute kidney injury, PREVENTIVE MEDICINE, PRIMARY CARE, QUALITATIVE RESEARCH, HEALTH SERVICES ADMINISTRATION & MANAGEMENT

SCHOLARONE™
Manuscripts

Implementation of 'sick day guidance' to prevent community-based Acute Kidney Injury: a qualitative study

Authors

Anne-Marie Martindale (corresponding author)

National Institute for Health Research Collaboration for Leadership in Applied Health Research and Care Greater Manchester, UK.

University of Manchester, 5th Floor, room 5.02, Williamson Building, Oxford Road, M13 9PL.

anne-marie.martindale@manchester.ac.uk

Rebecca Elvey

National Institute for Health Research Collaboration for Leadership in Applied Health Research and Care Greater Manchester, UK.

University of Manchester, 5th Floor, Suite 6, Williamson Building, Oxford Road, M13 9PL.

Rebecca.elvey@manchester.ac.uk

Sue Howard

National Institute for Health Research Collaboration for Leadership in Applied Health Research and Care Greater Manchester, UK.

Salford Royal NHS Foundation Trust, 3rd Floor, Mayo Building , Stott Lane, M6 8HD.

Susan.howard@srft.nhs.uk

Sheila McCorkindale

NHS Salford Clinical Commissioning Group, 7th Floor, St James's House Pendleton Way Salford, M6 5FW. sheila.mccorkindale@nhs.net

Smeeta Sinha

Salford Royal NHS Foundation Trust, Stott Lane, M6 8HD, smeeta.sinha@srft.nhs.uk

Tom Blakeman

For peer review only - http://bmjopen.bmj.com/site/about/guidelines.xhtml

National Institute for Health Research Collaboration for Leadership in Applied Health Research and Care Greater Manchester, UK.

University of Manchester, 6th Floor, Suite 6, Williamson Building, Oxford Road, M13 9PL.

tom.blakeman@manchester.ac.uk

Keywords

Acute Kidney Injury; Primary care; Qualitative; communication; patient safety

Word count 4153

For peer review only - http://bmjopen.bmj.com/site/about/guidelines.xhtml

Implementation of 'sick day guidance' to prevent community-based Acute Kidney Injury: a qualitative study

Abstract

Objectives: The study sought to examine the implementation of sick day guidance cards designed to prevent acute kidney injury (AKI), in primary care settings.

Design: Qualitative semi-structured interviews were conducted and comparative analysis informed by Normalisation Process Theory (NPT) was undertaken to understand sense-making, implementation and appraisal of the cards and associated guidance.

Setting: A single primary care health setting in the North of England.

Participants: 29 participants took part in the qualitative evaluation: 7 GPs, 5 practice nurses, 5 community pharmacists, 4 practice pharmacists, 2 administrators, 1 health care assistant, and 5 patients.

Intervention: The sick day guidance intervention was rolled out (2015-2016) in general practices (n=48) and community pharmacies (n=60). The materials consisted of a 'medicine sick day guidance' card, provided to patients who were taking the listed drugs. The card provided advice about medicines management during episodes of acute illness. An information leaflet was provided to healthcare practitioners and administrators suggesting how to use and give the cards.

Results: Implementation of sick day guidance cards to prevent AKI entailed a new set of working practises across primary care. A tension existed between ensuring reach in

For peer review only - http://bmjopen.bmj.com/site/about/guidelines.xhtml

administration of the cards to at risk populations whilst being confident to ensure patient understanding of their purpose and use. Communicating the concept of temporary cessation of medicines was a particular challenge and limited their administration to patient populations at higher risk of AKI, particularly those with less capacity to self-manage.

Conclusions: Sick day guidance cards that focus solely on medicines management may be of limited patient benefit without adequate resourcing, or if delivered as a standalone intervention. Development and evaluation of primary care interventions is urgently warranted to tackle the harm associated with AKI.

284 words

For peer review only

For peer review only - http://bmjopen.bmj.com/site/about/guidelines.xhtml

Strengths and limitations of this study

- Using Normalisation Process Theory (NPT) enabled a comprehensive understanding of the sense-making, use and appraisal of the AKI sick day card initiative.
- Interviews with a range of professionals (GPs, nurses, community and practice-based pharmacists, a health care assistant, practice administrators) and patients enhanced understanding of the individual and collective working practises surrounding the professional implementation AKI sick day guidance cards.
- Patient recruitment to the qualitative evaluation via general practice was slow and yielded only five patient-participants. This limited analysis of patient use of sick day guidance in everyday life.
- Learning suggests that alternative; community based methods of recruitment, such as through older persons organisations may yield higher numbers of patient-participants.

For peer review only - http://bmjopen.bmj.com/site/about/guidelines.xhtml

Introduction

Addressing the harm related to Acute Kidney Injury (AKI) is a worldwide priority.[1] AKI is characterised as a sudden reduction in kidney function over hours or days.[2-4] It is a marker of illness severity and is seen as a ‘force multiplier,’ complicating episodes of acute illness.[3] As a clinical syndrome, the majority of cases of AKI are due to a combination of underlying infection, hypovolaemia (low circulatory blood volume), hypotension (low blood pressure) and medication effects.[3] Addressing these potentially modifiable factors are central to both the prevention and management of AKI and its associated burden.[2-4]

Across the United Kingdom, patient safety initiatives have been established to address the morbidity, mortality and costs linked to AKI.[2 5-7] In Scotland, informed by findings from a primary care study conducted by NHS Highland, medicine sick day rules have been made available nationally through the Scottish Patient Safety Programme.[6 8] The introduction of medicine sick day rules relates to national guidance, published by the National Institute for Health and Care Excellence (NICE) as well as by the Royal College of Physicians of Edinburgh UK, which highlight a need to consider temporary cessation of medicines at times of acute illness.[4 9] That is, during these episodes, ‘any drug that reduces blood pressure, circulating volume, or renal blood flow’ increases the risk of AKI.[3] Medicines that exacerbate this risk include NSAIDS (non-steroidal anti-inflammatory drugs), diuretics, ACE inhibitors and angiotensin II receptor blockers (ARBs).[3] In addition, the Scottish medicine sick day rules refer to the temporary cessation of metformin, which may accumulate at times of reduced kidney function, resulting in an increased risk of adverse effects.[6] The NHS Scotland

For peer review only - http://bmjopen.bmj.com/site/about/guidelines.xhtml

'Medicine Sick Day Rules' cards provide instructions on temporarily stopping these specific types of medicines during episodes of acute illness.[6 8]

In England, within NHS England's Patient Safety Domain, the Think Kidneys Programme (https://www.thinkkidneys.nhs.uk) was established to tackle the harm associated with AKI.[10] Through the programme, resources have been developed, for primary and secondary care, including an Interim Position Statement on 'Sick Day' Guidance, which highlights a clinical equipoise surrounding the systematic implementation of sick day guidance.[11]

It was in this wider context that a Clinical Commissioning Group (CCG), in partnership with the local NHS Foundation Trust, embarked on service improvement initiatives to address the harm associated with AKI. Informed by the Scottish approach in conjunction with national guidance,[4 6 8] the CCG sought to implement the use of sick day guidance across general practices and community pharmacies within its boundaries. The Sick Day Guidance Project is outlined in Table 1 as well as Figures 1 and 2.

In accordance with NHS England Think Kidneys guidance, the project entailed formal evaluation. With a view to provide a platform for future larger scale evaluation, the study sought to explore processes surrounding the implementation of sick day guidance in primary care.

For peer review only - http://bmjopen.bmj.com/site/about/guidelines.xhtml

Table 1. The Sick Day Guidance Project TIDIER [12]

TIDieR Item	Brief Description
**Name**	Salford Kidney Implementation Project
**1 Why**	The Salford Partnership for Advancing Renal Care (SPARC) was established to ensure a shared strategy and optimise kidney care across the City.<br>The ambition of sick day guidance is to reduce the risk of avoidable harm to patients taking certain medications. Salford CCG in collaboration with SPARC defined the original implementation design of the sick day guidance intervention.
**2 What**	Medicines sick day guidance in two phases of work.
**3 Materials**	• Sick day guidance cards that suggested the temporary cessation of medicines during bouts of sickness were produced, the text was replicated from the NHS Highlands sick day rules card.<br>• Two, one and a half hour, educational events were run for healthcare professionals, organised and delivered by the Steering Group. This included why AKI is important from a local and national context.<br>• Information leaflet outlining the sick day guidance project and guidance on how to use the sick day guidance cards, and poster summarising this information for use in practice.<br>• Poster for patients promoting the sick day guidance card intervention to be used in waiting areas.
**4 Procedures**	1. Training was offered to all general practitioners, practice nurses and the wider practice team, and to community pharmacists for the sick day guidance card implementation.<br>2. During Phase One, the cards were distributed to all community pharmacies and general practices accompanied by an information leaflet and poster with patient engagement instructions. Distribution was carried out by project facilitators face to face, to explain and address any questions arising.<br>3. Two further face to face visits were made to each general practice and pharmacy by the NIHR CLAHRC GM project team to reinforce the project/provide additional materials/support.<br>4. The cards were provided to patients receiving the drugs listed on the card by general practices and community pharmacies.<br>5. Posters were displayed in practice waiting areas promoting the intervention to patients<br>6. General Practitioners and other practice staff were advised to record the intervention in Salford Integrated Records using Read code 80AG.<br>7. During Phase Two, the practice-based pharmacists accessed patient health records from Salford Royal NHS Foundation Trust to identify those at risk of AKI and constructed a database to record relevant data.<br>8. The practice-based pharmacists were to contact and educate patients on the sick day guidance and to issue a card. They were also expected to complete a medications review.<br>9. Approval was sought to ensure the project was in keeping with national Think Kidneys guidance.
**5 Who**	• The NIHR CLAHRC GM project team, (facilitation, project management, and research staff).<br>• The Steering Group (clinical, pharmacist and managerial staff at Salford CCG and Salford Royal NHS Foundation Trust, plus the NIHR CLAHRC GM project team).<br>• Salford CCG general practices and community pharmacies.
**6 How**	The initial recruitment of general practitioners onto the project was implemented via email, and then three face to face visits were delivered per practice/pharmacy by NIHR CLAHRC GM project team to ensure full understanding of the sick day guidance project. Support was also gained from the local pharmaceutical committee.
**7 Where**	General practices [48] and community pharmacies [60] in Salford. 106,000 cards were provided to general practices and community pharmacies for distribution.
**8 When and how much**	Cards provided to each patient, when they attended a general practice appointment or visited a pharmacy between March 2015 and January 2016.

For peer review only - http://bmjopen.bmj.com/site/about/guidelines.xhtml

	Practice pharmacists contacted patients who fit within their criteria for being at risk of AKI.
**9 Tailoring**	Whilst guidance on the explanation to give patients (described above) was provided, professionals were expected to use their professional judgement in deciding how to deliver the intervention.
**10 Modifications**	• Opportunistic observations were conducted during facilitation visits.<br>• Cards were noticed on pharmacy counters, which were available for anyone visiting the pharmacy to pick up and take.<br>• Practice pharmacists encountered difficulties around the process of completing the record searches and communicating with patients in that there was not enough time to do this, consequently, no face to face appointments took place and pharmacists tried to contact patients by telephone.<br>• One practice pharmacist developed their own AKI patient information sheet that was posted out with cards.
**11 How well (planned)**	Adherence and fidelity were not formally assessed, however, the facilitation visits were designed to provide flexible, on-going support and advice on delivering the intervention, and an understanding of how well the intervention was operating in practice was gained through these visits.
**12 How well (actual)**	Practice pharmacists encountered barriers to obtaining the information they needed.<br>• (CLAHRC facilitators gained understanding through their visits and the qualitative evaluation formally researched experiences of implementation – both these are documented in the report).<br>• Sustained efforts had to be made to recruit health professionals and patients via medical practices.

Figure 1 Sick day guidance card used during this project

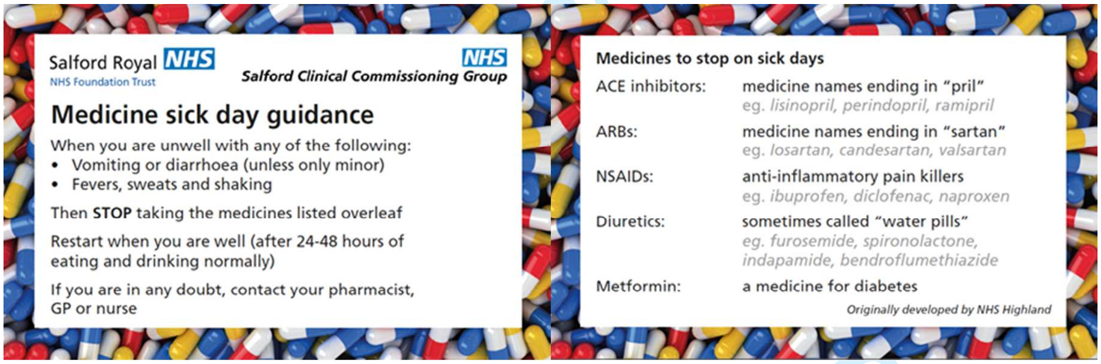

The NHS Highland sick day rules card was reproduced (Figure 1) with new logos.[6,8]

For peer review only - http://bmjopen.bmj.com/site/about/guidelines.xhtml

Figure 2 Guidance provided to health practitioners (shortened form)

MEDICINES AND DEHYDRATION: SICK DAY RULES

Offer the following information at the time of giving the card:

- Some medicines shouldn't be taken when you have an illness that makes you dehydrated. This is because they can either increase the risk of dehydration or because dehydration can lead to potentially serious side effects of the medicine.
- The medicine you are taking that falls into this category is [tell patient which medicine]
- Illnesses that can cause dehydration are vomiting, diarrhoea and fever.
- This advice does not apply to minor sickness or diarrhoea, which means a single episode
- If your medicines are in a blister pack you must take it to the chemists so the chemist can show you which ones you need to stop.
- If you have heart failure, you may stop these medicines for a maximum of 48 hours but after that you need to contact your GP or heart failure team for further advice.
- If your heart failure condition has been unstable recently then please contact the heart failure team for advice.

The list of medicines on the card is not exhaustive but they are highlighted because:

- Diuretics can cause dehydration or make dehydration more likely in an ill patient;
- ACE inhibitors, angiotensin II receptor blockers and NSAIDs may impair kidney function in a dehydrated patient, which could lead to kidney failure;
- Metformin dehydration increases the risk of lactic acidosis, a serious and potentially life-threatening side effect of metformin.

For peer review only - http://bmjopen.bmj.com/site/about/guidelines.xhtml

Methods

Study Design

A qualitative methodology informed by Normalisation Process Theory (NPT) was employed to examine the context, administration, interpretation and use of sick day guidance cards across a single primary healthcare setting in England. The approach to sampling, data collection and analysis centred on sense-making, experiences of implementation and appraisal of the AKI intervention in routine care delivery and everyday life.[13 14]

Data Sampling

To explore the trajectory of implementation across the CCG, all general practices (n = 48), community pharmacies (n = 60) and practice based pharmacists (n = 4) involved in the project were invited to take part in the evaluation. Information packs were provided to explain what involvement entailed. To facilitate patient participant engagement, general practices and community pharmacists were asked to provide information packs to patients who had received a card via a health practitioner. The final data sample of 29 interviews comprised: seven GPs; five practice nurses; five community pharmacists; four practice based pharmacists; two managers (one medical practice manager and one community pharmacy manager); and a health care assistant account.

Data Collection

Two qualitative researchers (AM-M; RE) conducted the 29 semi-structured interviews. These were conducted with participants across the CCG between June 2015 and April 2016. Interviews with the GPs, practice nurses, administrators and the health care assistant took

For peer review only - http://bmjopen.bmj.com/site/about/guidelines.xhtml

place in private locations within their general practices. Interviews with community pharmacists were also held at private locations at their places of work. Interviews with patients occurred at their homes. Interviews with three of the practice-based pharmacists took place at their place of work; one took place on the phone. The interviews ranged in length from 9 minutes to 66 minutes (median = 33 minutes). They were digitally audio-recorded in compliance with participants' consent and professionally transcribed.

Topics for the health practitioners included previous knowledge of AKI and involvement in kidney health initiatives, their role in the intervention, sense-making, and experiences of implementing and appraising the administration of sick day guidance cards. For patient-participant interviews, topics included: sense-making around health and illness; the context of card giving and guidance explanation; and comprehension and use of the guidance (Table 2).

For peer review only

Table 2. Summary Topic Guides for professional, managerial and support staff, and patient interviews

**Health professionals, managerial and support staff**	**Role in the AKI prevention project**<br>• Current role<br>• How supported patients to prevent AKI before the project?<br>• Preparation for role in sick day guidance/AKI project<br>• Specific training/education<br>• Additional needs for training/education in the area of AKI prevention	**Views of the AKI prevention project**<br>• Who offered sick day rules/other AKI interventions to? (types of patient)<br>• How did you engage with patients<br>• What works well and why? (enablers)<br>• What does not work well and why? (barriers)<br>• Views of its impact on patients<br>• Views of the impact on your work, and the rest of the healthcare team	**Integration with health care**<br>• How do sick day rules/other AKI initiatives, fit/link with other support for AKI prevention?<br>• Fit with long term conditions management and other health needs and services?<br>• How do they fit/link with hospital care/social/voluntary sector?<br>• Contact/interaction with the rest of the primary health care team, secondary care team(s) around sick day guidance/AKI more generally?<br>• Which health care professionals are best placed to provide AKI prevention support?
**Patients**	**Context/history**<br>• Length of time of condition/taking medicines<br>• Perceptions of health and illness in everyday life<br>• Management of medicines and/or acute episodes of illness before the project (whether used a sick day guidance before/blister packs)<br>• Difficulties experienced around managing medicines and any needs?	**The sick day guidance/other kidney health interventions**<br>• How they found out about the service?<br>• Whether used the card or not?<br>• What do they find useful or like about it?<br>• What do they not find useful or dislike about it?<br>• Do they feel it has helped them? If so, how?<br>• Could it be improved? If so, how?<br>• Which healthcare practitioners could/should provide the cards? (where and when)<br>• Who are sick day cards/other AKI interventions suitable for?	**Coordination of care**<br>• Who is involved in their care?<br>• How/where does the sick day guidance/other support provided as part of the project, fit with other services or care received or other self-care undertaken?

For peer review only

Data Analysis

A-M M and TB had access to the data and they conducted the analysis. As the data was qualitative in nature the decision was taken to store it securely and not to publish it as this could compromise participant anonymity. A-M M developed a thematic analysis framework using the evaluation objectives and the four core constructs of Normalisation Process Theory (NPT) to understand implementation.[13 14] NPT is concerned with social action rather than attitudes, to identify and understand the processes which constrain or enable the embedding and integration of a complex intervention into routine care. The four NPT constructs are coherence (sense-making), cognitive participation (relational work), collective action (operational work) and reflexive monitoring (appraisal).[13 14]

The questions asked of the health practitioner interview data included:

- how do they make sense of implementing the sick day card initiative?
- what work have they done to implement the initiative?
- how is the initiative being communicated or enacted by local others?
- what judgments have been made about the initiative?

The questions we asked of the patient participant data included:

- how does the participant make sense of health and illness?
- what was the context of the participant receiving a card and guidance?
- how did they make sense of the card and implement the guidance in their day to day lives?
- how did they value the intervention?

For peer review only - http://bmjopen.bmj.com/site/about/guidelines.xhtml

As the interviews were completed data from each one account was grouped according to role, which resulted in six data sets: GP; practice nurse and health care assistant; administration; community pharmacist; practice pharmacist; and patient-participant. Thematic analysis was carried out by A-M M and TB within and then across the six data sets to explore similarities and differences in context, sense-making, implementation and appraisal of the card.[15] Only certain members of the research team (TB, A-MM, RE) had access to the unpublished data (A-M M, RE and TB) and to materials developed at each stage of the analysis so as not to compromise participant anonymity.

Results

A version of the findings of this paper are included in a wider report that has been provided to the funding organisation[16]. Acute Kidney Injury (AKI) was viewed as a new phenomenon and the implementation of sick day guidance cards entailed a new set of working practises. Analysis indicated that AKI prevention guidance was not necessarily a straightforward concept to understand, or to communicate. Health practitioners thought the cards required some knowledge of illness symptoms and medicines, and that patients had to decide how severe the symptoms were before acting, or re-starting their medication. One practice pharmacist stated '*…patients don't understand what fever is…they think that if they've got a headache it's fever…we're trying to explain and they don't understand, or they say well, if I had a bout of diarrhoea do I stop the medication…it's severe. Well, what is severe, you know? Obviously it's very subjective…*' (SKHIP13PP).

For peer review only - http://bmjopen.bmj.com/site/about/guidelines.xhtml

Comparative analysis highlighted a tension between the need to achieve reach to the populations deemed at risk (i.e. those on relevant medicines) and at the same time ensure comprehension concerning use of the guidance. There was evidence that this tension influenced the implementation of the sick day guidance intervention. The following sections describe the different approaches employed.

Administration of the sick day guidance card in conjunction with face-to-face communication

A common theme was health professionals and patients valuing the need to explain the guidance in person. One patient reflected '*I don't think that it should be just put on a counter… I don't think, number one, they'll read it, number two, they'll digest what's on it, or number three, they'll apply it to themselves'* (SKHIP22PA). A practice nurse thought dialogue was also important to reduce miscommunication, avoid patient confusion and additional GP workload.

'I always explain …There's no point giving someone a card if they don't understand what it's for…my grandma wouldn't understand that. She'd probably misinterpret that and…stop taking everything' (SKHIP25PN).

Analysis of health practitioner and patient accounts revealed that patients responded to the guidance in a variety of ways, not always as intended. One patient participant used the terms sickness and illness interchangeably and spoke of different classifications of illness. She asked which type the guidance card was referring to, to be confident of following the instructions properly.

For peer review only - http://bmjopen.bmj.com/site/about/guidelines.xhtml

'What do you define as illness…? Well, I suppose I don't know… I've got arthritis, that's not an illness it's just a thing of life when you get older... I've had spinal surgery, but they're not illnesses…' (SKHIP22PA).

Two health practitioners reported instances of patients with medication associated diarrhoea stopping their tablets since receiving a card. This unintended consequence of the initiative, lead to those patients being prescribed alternative medication to alleviate the side effect. A couple of patient participant accounts revealed a lack of willingness to follow the guidance as it had not been implemented by their hospital specialist, whose opinion they trusted, and they did not want to make their condition worse. *'I'd rather feel sick than have a problem with the high blood pressure…'* (SKHIP31PA).

The concept of temporary cessation of medicines required careful consideration, for example when to stop, restart and what dosage to reinstate. *'We don't have enough data or…best practice… if you stop the metformin or whatever medication how long do you stop it for…? Then after a week are you going to restart them again on the ten milligram or are you going to start them on the 1.5, the 2.5…?*' (SKHIP14GP).

Although valued by the health practitioners interviewed, implementation of sick day guidance initiative demanded extra work. In general practice, this was deemed less problematic when it fitted into existing long-term condition review appointments, particularly with practice nurses or health care assistants. In community pharmacies, implementation sat more readily within face-to-face medication review appointments or

For peer review only - http://bmjopen.bmj.com/site/about/guidelines.xhtml

opportunistic over-the-counter interactions, including the purchase of non-steroidal anti-inflammatory drugs (NSAIDS, such as ibuprofen). One community pharmacist used the purchase of anti-diarrhoeal or sickness medications as an opportunity to administer AKI guidance.

'*…when people have been coming in to buy stuff for sickness or diarrhoea… If it turns out that they're on one of the medications that's on the card, then we'll give them a card then as well and explain about it*' (SKHIP5CP).

There were limits to the implementation of sick day guidance in patient populations deemed at increased risk of AKI. Concerns were expressed across the health professionals interviewed that the cards and temporary cessation of medications were not suitable for patients with cognitive impairments such as Alzheimer's disease, reduced literacy in English, those with advanced learning difficulties or visual impairments, or for elderly housebound patients taking multiple medicines. One community pharmacist commented on the difficulties facing patients and carers using dosette box (blister pack) systems.

'*they* (patients) *might have four or five tiny little white ones, and then if they're elderly or they can't see the markings, they don't know what tablet they should be stopping…. if it was a family member looking out for it, that would be I guess possible, but a lot of the carers are not allowed to alter any medication'* (SKHIP7CP).

For peer review only - http://bmjopen.bmj.com/site/about/guidelines.xhtml

Administration of sick day guidance cards to patients in conjunction with telephone consultations

Phase Two of the project entailed Practice Pharmacists supporting the implementation of the sick day guidance cards in general practices (see Table 1). All of the four CCG employed pharmacists valued and engaged with the project. However, they outlined difficulties fitting the implementation in with their pre-existing workload. There were more patients to work with than anticipated, and the searches, writing to patients, communicating with them and feeding the results back to GPs took longer to complete than the pharmacists described having time for.

To implement the project in this context, a decision was made to have telephone conversations with patients rather than face-to-face interactions. However, this created additional challenges. The phone calls took as long as the face-to-face encounters as the pharmacists expressed a professional need to do things '*properly*'. They reported patients not always being happy to talk with a perceived stranger on the phone about their health. Patient understanding was harder to assess and patients did not necessarily agree to enact the guidance if they became ill. Unlike the face-to-face GP and practice nurse consultations, patients on the other end of the phone had no prior trusting relationship with the practice pharmacist. One pharmacist tried to mitigate some of these issues by talking with a GP in advance of phoning:

'…I'm not going to just pick up the phone and ring this patient now, I'm going to ask the GP what he thinks… for the slightly elderly- some patients, perhaps mental health issues….They obviously know their patients much better than I do so I always take their advice' (SKHIP11PP).

For peer review only - http://bmjopen.bmj.com/site/about/guidelines.xhtml

The community pharmacists also spoke of the difficulties of assessing patient comprehension in this way. '*I've had to phone patients …if you've got a query or the prescription will be changed or we'll want to question something …sometimes they're on the ball, they completely know, and sometimes they're just so confused'* (SKHIP7CP).

Sick day guidance cards being administered without verbal or written communication

Instructions administered to health practitioners (Figure 2) stressed the need for dialogue with patients to check understanding. However, this did not always occur. Reasons included other work demands during a practice based consultation, limited time for dialogue, forgetting to discuss it, and some lack of confidence about what to say, partly because of the limited evidence base and so as not to confuse patients, especially those where less fluent in English '*we have quite a lot of different ethnicities here…they've got limited English I think they're not quite sure and it takes quite a while explaining …about what medicines to stop, when to stop it, when to restart it…*' (SKHIP10PN).

Though the community pharmacists were willing to talk with patients about the guidance cards, time shortages and other work demands impinged on implementation. One community pharmacist stated '*Half the time it's remembering to do it because you're thinking about that many different things*' (SKHIP5CP). In addition, they did not always have face-to-face contact with patients '*we've got like 900 of our own patients and we just make the packs and then send them out and delivery, so we don't actually have that much patient contact*' (SKHIP7CP).

For peer review only - http://bmjopen.bmj.com/site/about/guidelines.xhtml

Some health practitioners felt that the cards were self-explanatory. One practice nurse said '*vomiting is vomiting and diarrhoea is diarrhoea*' (SKHIP25PN). However, others did not agree. One GP thought it was really important to provide patients with written material to aid understanding and compliance '*with certain other sort of medicine regimes, we ask them to stop temporarily if there's a drug interaction and patients are okay with that, as long as you give them sort of written instructions and they know exactly why they're stopping. A lot of it is to do with the understanding. They don't like stopping things if they don't understand why…*' (SKHIP20GP).

A couple of patient accounts referred to finding cards in public information areas of medical practices and community pharmacists. One patient who found a card in this manner wanted to share the sick day guidance message '*…I went into the pharmacy last week, they were on the counter…I picked one up and brought it home …I think it's such a good idea that I've given one to my sister*' (SKHIP22PA).

Communication of AKI risk, but limited use of a sick day guidance card

One GP worked exclusively with patients in care homes across the CCG, which included patients who were diagnosed with cognition limiting conditions such as dementia. Though the guidance messages were deemed pertinent to these groups of patients more vulnerable to AKI, their use was limited due to a potential lack of understanding. *'So we have the card. We didn't use it a lot…We used it to give to the carers. I used it to give to a few of the patients that have capacity'* (SKHIP14GP).

For peer review only - http://bmjopen.bmj.com/site/about/guidelines.xhtml

The need for appropriate training for carers, nursing staff and associated social workers was raised, beyond the level of the sick day guidance card. Specifically there was felt to be an on-going need for health practitioners to highlight the importance of fluid management in conjunction with medicines management. *'…they* (dementia patients) *ended up not eating or drinking, worsening of the renal function and become unwell and they end up in hospital…' So it's working with the carer as well to understand…. It's serious things that they might die from, not being hydrated'* (SKHIP14GP).

Discussion

Principal Findings

Implementation of sick day guidance cards to prevent community based AKI entailed a new set of working practises. The temporary cessation of medicines during episodes of acute illness was not necessarily a straightforward concept to understand or communicate. Comparative analysis of participants' accounts highlighted a tension between ensuring reach in administration of the cards to at risk populations whilst being confident to ensure patient understanding of their purpose and use.

Strengths and Weaknesses of this study

Unlike an earlier study,[17] a key strength of this evaluation was to conduct an in-depth exploration of systematic roll out across a single healthcare setting. Use of Normalisation Process Theory ensured that a range of individual and collective working practises were considered during analysis.[13 14] This included exploring types of work undertaken in both

For peer review only - http://bmjopen.bmj.com/site/about/guidelines.xhtml

general practices and community pharmacies and their use by a range of health professionals in these different settings.

The study entailed comparative analysis of both patient and professional accounts in order to explore their use in clinical interactions as well as in everyday life. Though professional accounts allowed descriptions of experiences of use by patients, difficulties were encountered recruiting patient participants who had experiences of having used a sick day guidance card at times of acute illness. It is important to acknowledge that only five patients were interviewed in spite of extensive recruitment efforts. During the course of the interviews, health practitioners were asked about patient sense-making, use and appraisal of the guidance cards. In light of limited patient involvement these accounts became more important. We acknowledge that they are third order interpretations; our interpretations of what health practitioners reported about patients' sense-making, appraisal and use of the cards. However, the comparative approach taken has facilitated understanding of the pluralistic journeys of the cards and their intended and unintended messages and trajectories from card giver to patient across the 29 interviews. Future studies may benefit from sampling patients who have been coded in general practice as having been provided sick day guidance (i.e. Read Code 8OAG. 'Provision of information about Acute Kidney Injury')[18] and also who have been coded with an episode of acute illness (e.g. gastroenteritis, acute respiratory infection).

Comparison with other studies

In terms of professional responsibility, there are recognised boundaries to the role of general practitioners in supporting self-management.[19] The findings of this study resonate

For peer review only - http://bmjopen.bmj.com/site/about/guidelines.xhtml

and build on the results of previous research, which highlighted issues around the consistency of clinical message, and the additional work required to reduce the risk of harm from AKI using medicines management interventions.[17 20] The intervention was conducted at a time when concern was raised that UK general practice workload may be at 'saturation point.'[21] Results suggested that this influenced engagement with the CCG led initiative.

Though currently available through the Scottish Patient Safety Programme,[6] the findings from this qualitative study resonate with recently published literature, which highlights a need for a more robust evidence base surrounding the systematic implementation of sick day guidance cards.[22-24] A recent systematic review showed that 'there is no evidence of the impact of drug cessation interventions on AKI incidence during inter-current illness in primary or secondary care.'[22 24] In addition, results from a population-based cohort study indicate that patient co-morbidities including chronic kidney disease are much more strongly associated with AKI and that treatment with either an ACE Inhibitor or an ARB is only associated with a small increase in AKI risk.[23] That is, younger patients with limited comorbidity (e.g. on ACEI for treatment of hypertension) have a low absolute risk of AKI, whilst patients living with multi-morbidity in whom there may be professional concerns about ensuring effective risk communication, have a much higher risk of AKI.[23]

Implications for clinicians, policy makers and future research

In the UK, NICE recommends raising awareness of AKI in higher risk population groups with specific reference to patients who: have existing CKD; have had a previous episode of illness complicated by AKI; and/or have neurological or cognitive impairment and who may be reliant on carers for support with fluid intake during an acute illness (e.g. those with

For peer review only - http://bmjopen.bmj.com/site/about/guidelines.xhtml

For peer review only

cognitive impairment).[25] This may help address a knowledge gap in patient and public understanding of the importance in the maintenance of kidney health. A survey conducted in 2014 on behalf of NHS England indicated that ‘about half of the population in Great Britain don’t think their kidneys make urine’ and ‘only an eighth (12%) of interviewees thought their kidneys had a role in processing medicines.’[26] However, the findings from this study suggest an evidence base is urgently warranted to determine how best to resource effective self-management support for higher risk patient populations.

The NHS England Urgent and Emergency Care Review also emphasised the need for better support for people to self-care.[27] Our analysis in conjunction with the research by Mansfield et al (2016),[23] suggest sick day guidance cards alone, that focus solely on temporary cessation of medicines, are unlikely to be sufficient to reduce the harm associated with AKI. Specifically, the findings suggest other strategies may need to be resourced to prevent AKI in people with complex health and social care needs such as those living with dementia. A key issue raised was to provide better education and support for carers (both professional and informal). The Royal College of General Practitioners has provided guidance on the development of ‘carer friendly’ practices and the establishment of Patient Participation Groups, may be a mechanism to resource and integrate support for carers into the organisation of acute care.[28 29]

Conclusions

The findings from this qualitative evaluation suggest that there are boundaries to the implementation of sick day guidance cards to prevent Acute Kidney Injury in primary care. A common theme was the need to ensure patient understanding of their purpose and use.

For peer review only - http://bmjopen.bmj.com/site/about/guidelines.xhtml

Communicating the concept of temporary cessation of medicines was a particular challenge and limited their administration to patient populations at higher risk of AKI, particularly those with less capacity to self-manage. The analysis suggests that sick day guidance cards that focus solely on medicines management may be of limited benefit without either adequate resourcing, or if delivered as a standalone intervention. Development and evaluation of a primary care intervention encompassing a range of initiatives to tackle the harm associated with AKI is warranted.

Declarations

Ethics approval and consent to participate

Ethical approval was gained from Leeds West Research Ethics Committee (REC Reference Number: 15/YH/0174). Informed consent was gained from all participants prior to interview.

Consent for publication

Not applicable.

Availability of data and material

The data has been stored securely with password protected files to ensure confidentiality, in keeping with the research protocol and good data management guidelines. It will not be shared.

Competing interests

The authors declare that they have no competing interests.

Funding

For peer review only - http://bmjopen.bmj.com/site/about/guidelines.xhtml

This project was funded by the National Institute for Health Research Collaboration for Leadership in Applied Health Research and Care (NIHR CLAHRC) Greater Manchester and NHS Salford CCG. The NIHR CLAHRC Greater Manchester is a partnership between providers and commissioners from the NHS, industry and the third sector, as well as clinical and research staff from the University of Manchester. The views expressed in this article are those of the authors and not necessarily those of the NHS, NIHR or the Department of Health".

Authors Contributions

TB, RE, SS, SM and SH conceived and designed the study, A-MM and RE collected the data, TB and A-MM analysed the data. All authors contributed to writing the manuscript and have read and approved the final manuscript.

Acknowledgements

The authors would like to thank the project steering group for their input and guidance throughout the study.

Authors' information (optional)

TB is a member of NHS England's Think Kidneys Programme Board.

For peer review only - http://bmjopen.bmj.com/site/about/guidelines.xhtml

References

1. Mehta RL, Cerdá J, Burdmann EA, et al. International Society of Nephrology's 0by25 initiative for acute kidney injury (zero preventable deaths by 2025): a human rights case for nephrology. *The Lancet*;385(9987):2616-43.
2. Kidney Disease Improving Global Outcomes (KDIGO) Acute Kidney Injury Work Group. KDIGO Clinical Practice Guideline for Acute Kidney Injury. *Kidney International Supplement* 2012;**2**(1):1–138.
3. NHS England, UK Renal Registry, Think Kidneys. Acute Kidney Injury Best Practice Guidance: Responding to AKI Warning Stage Test Results for Adults in Primary Care: Think Kidneys, 2016.
4. National Institute for Health and Care Excellence. Acute Kidney Injury: prevention, detection and management (CG169): NICE, 2013.
5. NHS England, UK Renal Registry, Think Kidneys. Acute Kidney Injury Warning Alert Best Practice document: Think Kidneys, 2014.
6. NHS Scotland, Health Improvement Scotland, Scottish Patient Safety Programme. Medicine Sick Day Rules Cards: NHS Scotland, Health Improvement Scotland; 2016 [Availablefrom: http://www.scottishpatientsafetyprogramme.scot.nhs.uk/programmes/primary-care/medicine-sick-day-rules-card accessed 4th of April, 2017.
7. Kerr M, Bedford M, Matthews B, et al. The economic impact of acute kidney injury in England. *Nephrology Dialysis Transplantation* 2014;29(7):1362-68.
8. Morrison C. Medicine Sick Day Rules Cards - Intermin Evaluation. In: Wilson M, ed.: NHS Highland, 2014.
9. Feehally J, Gilmore I, Barasi S, et al. RCPE UK consensus conference statement: management of acute kidney injury: the role of fluids, e-alerts and biomarkers. *J R Coll Physicians Edinb* 2013;43 doi: 10.4997/jrcpe.2013.109
10. NHS England, UK Renal Registry. Think Kidneys [http://www.thinkkidneys.nhs.uk/] Accessed 7 Apr 2015.
11. NHS England, UK Renal Registry, Think Kidneys. “Sick day” guidance in patients at risk of Acute Kidney Injury: an Interim Position Statement from the Think Kidneys Board: Think Kidneys, 2015.
12. Hoffmann TC, Glasziou PP, Boutron I, et al. Better reporting of interventions: template for intervention description and replication (TIDieR) checklist and guide. *BMJ* 2014;348 doi: 10.1136/bmj.g1687
13. May C, Mair F, Finch T, et al. Development of a theory of implementation and integration: normalisation process theory. *Implement Sci* 2009;4
14. Murray E, Treweek S, Pope C, et al. Normalisation process theory: a framework for developing, evaluating and implementing complex interventions. *BMC Med* 2010;8:63.
15. Silverman S. Interpreting Qualitative Data: Methods for Analysing Talk, Text and Interaction. London: Thousand Oaks 2001.
16. NIHR Collaboration for Leadership in Applied Health Research and Care Greater Manchester. Salford Clinical Commissioning Group Sick Day Guidance Project: Final Report. Manchester: NIHR CLAHRC GM, 2016.
17. Morris RK AD, Phipps D, Bower P, O'Donoghue D, Roderick P, Harding S, Lewington A, Blakeman T. Preventing Acute Kidney Injury: A qualitative study exploring 'sick day rules' implementation in primary care. *BMC Family Practice* 2016;FAMP-D-15-00010R3 (Accepted for Publication 12 July 2016)

For peer review only

For peer review only - http://bmjopen.bmj.com/site/about/guidelines.xhtml

18. NHS North West Commissiong Support Unit. Salford Standards Read Code Dictionary. Specification for Read V2 coding for: Salford Clinical Commissioning Group: NHS North West Commissioning Support Unit, 2016.
19. Blakeman T, Macdonald W, Bower P, et al. A qualitative study of GPs' attitudes to self-management of chronic disease. *Br J Gen Pract* 2006;56(527):407-14.
20. Phipps DL, Morris RL, Blakeman T, et al. What is involved in medicines management across care boundaries? A qualitative study of healthcare practitioners' experiences in the case of acute kidney injury. *BMJ Open* 2017;7(1) doi: 10.1136/bmjopen-2016-011765
21. Hobbs FDR, Bankhead C, Mukhtar T, et al. Clinical workload in UK primary care: a retrospective analysis of 100 million consultations in England, 2007-14. *The Lancet* 2016; 287 (10035): 2323-2330.
22. Morden A, Horwood J, Whiting P, et al. The risks and benefits of patients temporarily discontinuing medications in the event of an intercurrent illness: a systematic review protocol. *Systematic Reviews* 2015;4(1):1-6. doi: 10.1186/s13643-015-0135-y
23. Mansfield KE, Nitsch D, Smeeth L, et al. Prescription of renin–angiotensin system blockers and risk of acute kidney injury: a population-based cohort study. *BMJ Open* 2016;6(12) doi: 10.1136/bmjopen-2016-012690
24. Whiting P MA, Tomlinson LA, Caskey F, Savovic J, Blakeman T, Tomson C, Stone T, Richards A, Horwood J. What are the risks and benefits of temporarily discontinuing medications to prevent acute kidney injury? A Systematic Review and meta-analysis. *BMJOpen* 2017;bmjopen-2016-012674.R1 Accepted for Publication 25th October, 2016
25. National Institue for Health and Care Excellence. Acute kidney injury Quality standard nice.org.uk/guidance/qs76: NICE, 2014.
26. NHS England, UK Renal Registry, Think Kidneys. Understanding what the public know about their kidneys and what they do. Findings from Ipsos MORI survey – July 2014: Think Kidneys, 2014.
27. NHS England. High quality care for all, now and for future generations: Transforming urgent and emergency care services in England - Urgent and Emergency Care Review. Leeds: Urgent and Emergency Care Review Team, NHS England, 2013.
28. Royal College of General Practitioners. RCGP Supporting Carers in General Practice: Summary report on GP practice journeys towards improved carer identification and support. London: Royal College of General Practitioners, 2013.
29. NHS Employers. 2015/16 General Medical Services (GMS) contract: Guidance for GMS contract 2015/16: NHS Employers, 2015.

For peer review only

For peer review only - http://bmjopen.bmj.com/site/about/guidelines.xhtml

Implementation of 'sick day guidance' to prevent community-based Acute Kidney Injury: a qualitative study

*It has not been possible to identify responses to each guide question within the paper as some questions are not relevant to the text and to remain within the word length, however we have adhered to good ethical practice at all times.

COREQ Table

Guide question		Response	Manuscript page number
1	Interviewer/facilitator	The interviews were conducted by AM-M and RE	10
2	Credentials	AM-M Ba, Ma, PhD<br>RE Ba, Ma, PhD<br>SH BSc, PhD<br>SM MBChB<br>SS MBChB MRCP(UK) PhD<br>TB MBChB; PhD	1
3	Occupation	A-M M Post-doctoral research associate, Collaboration for Leadership in Applied Health Research and Care Greater Manchester) (CLAHRC GM),<br>RE Research Fellow, CLAHRC GM,<br>SH Kidney Research Programme Manager, CLAHRC GM<br>SS Senior renal consultant<br>TB Clinical Senior Lecturer and GP, CLAHRC GM.	1
4	Gender	A-M M female<br>RE female<br>SH female<br>SM female<br>SS female<br>TB male	*
5	Experience and training	A-M M is a social anthropologist by training and has had extensive training and experience conducting qualitative research<br>RE is an experienced and trained qualitative researcher in health<br>TB is an experienced and trained qualitative health researcher<br>SH, SS and SM all contributed to the design of the initiative and the evaluation.	*
6	Relationship established	The researchers conducting the interviews (A-M M, RE) did not know any of the interviewees prior to the research.	*
7	Participant knowledge of the researcher	Via information sheets and verbal clarification the participants knew that A-M M and RE were researchers evaluating the acute kidney injury (AKI) sick day guidance initiative.	10
8	Researcher characteristics	The researchers were working on a (CLAHRC) programme of research on kidney ill-health prevention in greater Manchester.	10

For peer review only

9	Methodological orientation and theory	We used normalisation process theory (NPT) which aims to explore the contexts, sense-making, activity and participant appraisal of an implementation project.	10, Table 2 (page 12), 13
10	Sampling	A combination of purposive and snowballing. Each pharmacy and GP practice in the locality was involved in the intervention, all were asked to take part in the evaluation and to help recruit patient participants via dissemination of information packs. We asked those we interviewed if they knew others who might want to take part.	10
11	Method of approach	Participants were contacted by email and post.	*
12	Sample size	29 participants	10
13	Non-participation	Not applicable	
14	Setting of data collection	Data were collected at the participants' place of work and over the phone	11
15	Presence of non-participants	Not applicable	
16	Description of sample		10-11
17	Interview guide	See Table 2	12
18	Repeat interviews	Not applicable	
19	Audio/visual recording	Audio recorded, with consent from the participant, as explained in verbal and written information	11
20	Field notes	Written after each interview and used to inform analysis	*
21	Duration	from 9 minutes to 66 minutes (median = 33 minutes)	11
22	Data saturation	participants were recruited until no new knowledge emerged	*
23	Transcripts returned	Participants were asked if they wanted a copy of the transcript, none did.	*
24	Number of data coders	2, Data analysis was conducted by A-M M and TB.	13
25	Description of the coding tree	Not relevant, analysis was focused around NPT and the evaluation objectives	14
26	Derivation of themes	Analysis was focused around the four constructs of NPT coherence (sense-making), cognitive participation (relational work), collective action (operational work) and reflexive monitoring (appraisal); the evaluation objectives and we allowed for serendipitous findings.	13-14
27	Software	Ms Word (transcripts, and fieldnotes) and Olympus software audio recordings were utilised repeatedly	*
28	Participant checking	Participants were not asked to provide feedback on the findings.	*
29	Quotations presented	Quotations are presented throughout the results section.	14-20
30	Data and findings consistent	The themes arising stem from the data and are consistent throughout.	14-20
31	Clarity of major themes	The major themes are presented under headings and under the heading Principal Findings	14-20, 21-22
32	Clarity of minor themes	Not applicable, there was no room within the word limit to discuss minor themes.	

For peer review only

BMJ Open

Understanding the implementation of 'sick day guidance' to prevent Acute Kidney Injury across a primary care setting in England: a qualitative evaluation

Journal:	*BMJ Open*
Manuscript ID	bmjopen-2017-017241.R1
Article Type:	Research
Date Submitted by the Author:	05-Jul-2017

Complete List of Authors:	Martindale, Anne-Marie; National Institute for Health Research Collaboration for Leadership in Applied Health Research and Care Greater Manchester; The University of Manchester, Centre for Primary Care, 5th Floor, room 5.02, Williamson Building, Oxford Road, M13 9PL. Fax 0161 275 7600<br>Elvey, Rebecca ; National Institute for Health Research Collaboration for Leadership in Applied Health Research and Care Greater Manchester; The University of Manchester, 5th Floor, Suite 6, Williamson Building, Oxford Road, M13 9PL.<br>Howard, Susan; National Institute for Health Research Collaboration for Leadership in Applied Health Research and Care Greater Manchester; Salford Royal NHS Foundation Trust, 3rd Floor, Mayo Building, Stott Lane, M6 8HD.<br>McCorkindale, Sheila; NHS Salford Clinical Commissioning Group, 7th Floor, St James's House Pendleton Way Salford, M6 5FW<br>Sinha, Smeeta; Salford Royal NHS Foundation Trust, Stott Lane, M6 8HD<br>Blakeman, Tom; National Institute for Health Research Collaboration for Leadership in Applied Health Research and Care Greater Manchester; The University of Manchester, 6th Floor, Suite 6, Williamson Building, Oxford Road, M13 9PL

**Primary Subject Heading**:	General practice / Family practice
Secondary Subject Heading:	Communication, Qualitative research
Keywords:	Acute kidney injury, PREVENTIVE MEDICINE, PRIMARY CARE, QUALITATIVE RESEARCH, patient safety

SCHOLARONE™ Manuscripts

Understanding the implementation of 'sick day guidance' to prevent Acute Kidney Injury across a primary care setting in England: a qualitative evaluation

Authors

Anne-Marie Martindale (corresponding author)

National Institute for Health Research Collaboration for Leadership in Applied Health Research and Care, Greater Manchester, UK.

University of Manchester, 5th Floor, room 5.02, Williamson Building, Oxford Road, Manchester, UK, M13 9PL. Fax 0161 275 7600.

Telephone 0161 275 7601

anne-marie.martindale@manchester.ac.uk

Rebecca Elvey

National Institute for Health Research Collaboration for Leadership in Applied Health Research and Care, Greater Manchester, UK.

University of Manchester, 5th Floor, Suite 6, Williamson Building, Oxford Road, Manchester, UK, M13 9PL. Rebecca.elvey@manchester.ac.uk

Susan J Howard

National Institute for Health Research Collaboration for Leadership in Applied Health Research and Care, Greater Manchester, UK.

Salford Royal NHS Foundation Trust, 3rd Floor, Mayo Building, Stott Lane, Salford, UK, M6 8HD. Susan.howard@srft.nhs.uk

Sheila McCorkindale

NHS Salford Clinical Commissioning Group, 7th Floor, St James's House Pendleton Way, Salford, UK, M6 5FW. sheila.mccorkindale@nhs.net

Smeeta Sinha

For peer review only - http://bmjopen.bmj.com/site/about/guidelines.xhtml

Salford Royal NHS Foundation Trust, Stott Lane, Salford, UK, M6 8HD.

smeeta.sinha@srft.nhs.uk

Tom Blakeman

National Institute for Health Research Collaboration for Leadership in Applied Health Research and Care, Greater Manchester, UK.

University of Manchester, 6th Floor, Suite 6, Williamson Building, Oxford Road, Manchester, UK, M13 9PL. tom.blakeman@manchester.ac.uk

Keywords

Acute Kidney Injury; Preventative medicine, Primary care; Qualitative research; patient safety

Revised word count 5008 (excluding title page, references, figures and tables)

For peer review only

Understanding the implementation of 'sick day guidance' to prevent Acute Kidney Injury across a primary care setting in England: a qualitative evaluation.

Abstract

Objectives: The study sought to examine the implementation of sick day guidance cards designed to prevent acute kidney injury (AKI), in primary care settings.

Design: Qualitative semi-structured interviews were conducted and comparative analysis informed by Normalisation Process Theory (NPT) was undertaken to understand sense-making, implementation and appraisal of the cards and associated guidance.

Setting: A single primary care health setting in the North of England.

Participants: 29 participants took part in the qualitative evaluation: 7 GPs, 5 practice nurses, 5 community pharmacists, 4 practice pharmacists, 2 administrators, 1 health care assistant, and 5 patients.

Intervention: The sick day guidance intervention was rolled out (2015-2016) in general practices (n=48) and community pharmacies (n=60). The materials consisted of a 'medicine sick day guidance' card, provided to patients who were taking the listed drugs. The card provided advice about medicines management during episodes of acute illness. An information leaflet was provided to healthcare practitioners and administrators suggesting how to use and give the cards.

For peer review only

Results: Implementation of sick day guidance cards to prevent AKI entailed a new set of working practises across primary care. A tension existed between ensuring reach in administration of the cards to at risk populations whilst being confident to ensure patient understanding of their purpose and use. Communicating the concept of temporary cessation of medicines was a particular challenge and limited their administration to patient populations at higher risk of AKI, particularly those with less capacity to self-manage.

Conclusions: Sick day guidance cards that focus solely on medicines management may be of limited patient benefit without adequate resourcing, or if delivered as a standalone intervention. Development and evaluation of primary care interventions is urgently warranted to tackle the harm associated with AKI.

284 words

For peer review only

Strengths and limitations of this study

- Using Normalisation Process Theory (NPT) enabled a comprehensive understanding of the sense-making, use and appraisal of the AKI sick day card initiative.
- Interviews with a range of professionals (GPs, nurses, community and practice-based pharmacists, a health care assistant, practice administrators) and patients enhanced understanding of the individual and collective working practises surrounding the professional implementation AKI sick day guidance cards.
- Patient recruitment to the qualitative evaluation via general practice was slow and yielded only five patient-participants. This limited analysis of patient use of sick day guidance in everyday life.
- Future study design would benefit from greater alignment between quantitative and qualitative elements of an evaluation

For peer review only

For peer review only - http://bmjopen.bmj.com/site/about/guidelines.xhtml

Introduction

Addressing the harm related to Acute Kidney Injury (AKI) is a worldwide priority.[1] AKI is characterised as a sudden reduction in kidney function over hours or days.[2-4] It is a marker of illness severity and is seen as a 'force multiplier,' complicating episodes of acute illness.[3] As a clinical syndrome, the majority of cases of AKI are due to a combination of underlying infection, hypovolaemia (low circulatory blood volume), hypotension (low blood pressure) and medication effects.[3] Addressing these potentially modifiable factors are central to both the prevention and management of AKI and its associated burden.[2-4]

Across the United Kingdom, patient safety initiatives have been established to address the morbidity, mortality and costs linked to AKI.[2 5-7] In Scotland, informed by findings from a primary care study conducted by NHS Highland, medicine sick day rules have been made available nationally through the Scottish Patient Safety Programme.[6 8] The introduction of medicine sick day rules relates to NHS Scotland Polypharmacy Guidance as well as national guidance, published by the National Institute for Health and Care Excellence (NICE) and by the Royal College of Physicians of Edinburgh UK. These publications highlight a need to consider temporary cessation of medicines at times of acute illness.[4 9 10]That is, during these episodes, 'any drug that reduces blood pressure, circulating volume, or renal blood flow' increases the risk of AKI.[3] Medicines that exacerbate this risk include NSAIDS (non-steroidal anti-inflammatory drugs), diuretics, ACE inhibitors and angiotensin II receptor blockers (ARBs).[3] In addition, the Scottish medicine sick day rules refer to the temporary cessation of metformin, which may accumulate at times of reduced kidney function, resulting in an increased risk of adverse effects.[6] The NHS Scotland 'Medicine Sick Day Rules' cards were

For peer review only - http://bmjopen.bmj.com/site/about/guidelines.xhtml

developed through extraction of NHS Scotland Polypharmacy Guidance (2012) and were 'designed with input from pharmacists, doctors and patients.' [10 11] They provide instructions on temporarily stopping these specific types of medicines during episodes of acute illness.[6 8]

In England, within NHS England's Patient Safety Domain, the Think Kidneys Programme (https://www.thinkkidneys.nhs.uk) was established to tackle the harm associated with AKI.[12] Through the programme, resources have been developed for primary and secondary care, including an Interim Position Statement on 'Sick Day' Guidance, which highlights a clinical equipoise surrounding the systematic implementation of sick day guidance. [13]

It was in this wider context that a Clinical Commissioning Group (CCG), in partnership with the local hospital, embarked on service improvement initiatives to address the harm associated with AKI. Informed directly by the Scottish approach in conjunction with national guidance[4 6 8] the CCG sought to implement the use of sick day guidance across general practices and community pharmacies within its boundaries. The Sick Day Guidance Project including an overview of the organisation of primary health care in England is outlined in Table 1 as well as Figures 1 and 2. In accordance with NHS England Think Kidneys guidance, the project entailed formal evaluation. With a view to provide a platform for future larger scale evaluation, the study sought to explore and understand processes underpinning the implementation of sick day guidance in primary care.

For peer review only - http://bmjopen.bmj.com/site/about/guidelines.xhtml

Table 1. The Sick Day Guidance Project TIDIER[14]

TIDieR Item	Brief Description
**Name**	Salford Kidney Implementation Project
**1 Why**	The Salford Partnership for Advancing Renal Care (SPARC) was established to ensure a shared strategy and optimise kidney care across the City.<br>The ambition of sick day guidance is to reduce the risk of avoidable harm to patients taking certain medications. Salford CCG in collaboration with SPARC defined the original implementation design of the sick day guidance intervention.<br>NIHR Collaboration for Leadership in Applied Health Research and Care (CLAHRC) Greater Manchester works in partnership with Salford CCG to support implementation and evaluation of projects. NIHR CLAHRC Greater Manchester evaluated this CCG priority and supported the implementation of sick day guidance.
**2 What**	Medicines sick day guidance in two phases of work.
**3 Materials**	• Sick day guidance cards that suggested the temporary cessation of medicines during bouts of sickness were produced, the text was replicated from the NHS Highland sick day rules card.<br>• Two, one and a half hour, educational events were run for healthcare professionals, organised and delivered by the Steering Group. This included why AKI is important from a local and national context.<br>• Information leaflet outlining the sick day guidance project and guidance on how to use the sick day guidance cards, and poster summarising this information for use in practice.<br>• Poster for patients promoting the sick day guidance card intervention to be used in waiting areas.
**4 Procedures**	1. Training was offered to all general practitioners, practice nurses and the wider practice team, and to community pharmacists for the sick day guidance card implementation.<br>2. During Phase One, the cards were distributed to all community pharmacies and general practices accompanied by an information leaflet and poster with patient engagement instructions. Distribution was carried out by project facilitators face to face, to explain and address any questions arising.<br>3. Two further face to face visits were made to each general practice and pharmacy by the NIHR CLAHRC GM project team to reinforce the project/provide additional materials/support.<br>4. The cards were to be provided to patients receiving the drugs listed on the card by general practices and community pharmacies.<br>5. Posters were displayed in practice waiting areas promoting the intervention to patients<br>6. General Practitioners and other practice staff were advised to record the intervention in Salford Integrated Records using Read code 80AG.<br>7. During Phase Two, the practice-based pharmacists accessed patient health records from Salford Royal NHS Foundation Trust to identify those at risk of AKI and constructed a database to record relevant data.<br>8. The practice-based pharmacists were to contact and educate patients on the sick day guidance and to issue a card. They were also expected to complete a medications review.<br>9. Approval was sought to ensure the project was in keeping with national Think Kidneys guidance.
**5 Who**	• The NIHR CLAHRC GM project team, (facilitation, project management, and research staff).<br>• The Steering Group (clinical, pharmacist and managerial staff at Salford CCG and Salford Royal NHS Foundation Trust, plus the NIHR CLAHRC GM project team).<br>• Salford CCG general practices and community pharmacies.
**6 How**	The initial recruitment of general practitioners onto the project was implemented via email, and then three face to face visits were delivered per practice/pharmacy by NIHR CLAHRC GM project team to ensure full understanding of the sick day guidance project. Support was also gained from the local pharmaceutical committee.
**7 Where**	General practices [48] and community pharmacies [60] in Salford. 106,000 cards were

For peer review only - http://bmjopen.bmj.com/site/about/guidelines.xhtml

	provided to general practices and community pharmacies for administration to patients.<br><br>In England, there were structural changes to the health service in 2013 and clinical commissioning groups (CCGs) were formed. Each CCG covers the population of a defined area (that is, patients registered at general practices within the area) and is responsible for planning and commissioning the majority of health services in that area. Primary health care services are provided by general practitioners (GPs) community pharmacies, dentists and opticians. Patients register with a GP practice and attend that practice for appointments with a GP(s). Community pharmacies, also known as local chemist shops, are found on most local high streets, in shopping centres and also in many large supermarkets. Community pharmacies dispense prescription medicines, sell other (non-prescription) medicines and various other goods (typically health-related, baby and cosmetic products) and also provide other services, such as medicines use reviews (MURs). Patients do not register with a community pharmacy and may use any pharmacy (for dispensing or other services), although many patients become regular users of their local pharmacy. Pharmacists also work in general practices; such 'practice-based' pharmacists review medicines prescribing and take part in projects, such as the 'sick day guidance' intervention described here.
**8 When and how much**	Cards were to be provided to a patient, when they attended a general practice appointment or visited a pharmacy between March 2015 and January 2016.<br>Practice pharmacists contacted patients who fit within their criteria for being at risk of AKI.
**9 Tailoring**	Whilst guidance on the explanation to give patients (described above) was provided, professionals were expected to use their professional judgement in deciding how to deliver the intervention.
**10 Modifications**	• Opportunistic observations were conducted during facilitation visits.<br>• Cards were noticed on pharmacy counters, which were available for anyone visiting the pharmacy to pick up and take.<br>• Practice pharmacists encountered difficulties around the process of completing the record searches and communicating with patients in that there was not enough time to do this, consequently, no face to face appointments took place and pharmacists tried to contact patients by telephone.<br>• One practice pharmacist developed their own AKI patient information sheet that was posted out with cards.
**11 How well (planned)**	Adherence and fidelity were not formally assessed, however, the facilitation visits were designed to provide flexible, on-going support and advice on delivering the intervention, and an understanding of how well the intervention was operating in practice was gained through these visits.
**12 How well (actual)**	Practice pharmacists encountered barriers to obtaining the information they needed.<br>• (CLAHRC facilitators gained understanding through their visits and the qualitative evaluation formally researched experiences of implementation – both these are documented in the CCG report).<br>• Sustained efforts had to be made to recruit health professionals and patients via medical practices.

For peer review only - http://bmjopen.bmj.com/site/about/guidelines.xhtml

Figure 1 Sick day guidance card used during this project

The NHS Highland sick day rules card was reproduced (Figure 1) with new logos.[6 8]

For peer review only

For peer review only - http://bmjopen.bmj.com/site/about/guidelines.xhtml

Figure 2 Guidance provided to health practitioners (shortened form)

For peer review only

For peer review only - http://bmjopen.bmj.com/site/about/guidelines.xhtml

Methods

Study Design

Aligned with the project objectives, Normalisation Process Theory (NPT) provided a sensitising framework to inform the topic guide and explore the context, administration, interpretation and use of sick day guidance cards across a single primary healthcare setting in England. [15,16] NPT is a theory of implementation developed through an in-depth analysis of chronic illness care in general practice. [15] It is a sociological theory that provides a structure to explore the individual and group work that people do surrounding the implementation of a complex intervention.[15,16,17]

Data Sampling

To explore the trajectory of implementation across the CCG, all general practices (n = 48), community pharmacies (n = 60) and practice based pharmacists (n = 4) involved in the project were invited to take part in the evaluation. Information packs were provided to explain what involvement entailed. To facilitate patient participant engagement, general practices and community pharmacists were asked to provide information packs to patients who had received a card via a health practitioner. The final data sample of 29 interviews comprised: seven GPs; five practice nurses; five community pharmacists; four practice based pharmacists; two managers (one medical practice manager and one community pharmacy manager); and a health care assistant, a person qualified to carry out routine health care tasks.

Data Collection

For peer review only - http://bmjopen.bmj.com/site/about/guidelines.xhtml

Two qualitative researchers (AM-M; RE) conducted the 29 semi-structured interviews. These were conducted with participants across the CCG between June 2015 and April 2016. Participants received an approved participant information sheet and consent form via post or email. Both were read by the researcher prior to interview and participants had the opportunity to ask questions, and have them answered satisfactorily. Informed consent was gained before each interview. Interviews with the GPs, practice nurses, administrators and the health care assistant took place in private locations within their general practices. Interviews with community pharmacists were also held at private locations at their places of work. Interviews with patients occurred at their homes. Interviews with three of the practice-based pharmacists took place at their place of work; one took place on the phone. The two researchers did not know any of the participants prior to interview. The interviews ranged in length from 9 minutes to 66 minutes (median = 33 minutes). They were digitally audio-recorded in compliance with participants' consent and professionally transcribed.

Interview topic guides were developed to explore the work being undertaken by professionals and patients surrounding the use of sick day guidance cards. NPT was used to inform the areas of questioning.[16] Topics for the health practitioners included previous knowledge of AKI and involvement in kidney health initiatives, their role in the intervention, sense-making, and experiences of implementing and appraising the administration of sick day guidance cards. For patient-participant interviews, topics included: sense-making around health and illness; the context of card giving and guidance explanation; and comprehension and use of the guidance (Table 2). Field notes about the encounter were written immediately after leaving the interview site and used to inform the analysis.

For peer review only - http://bmjopen.bmj.com/site/about/guidelines.xhtml

Participants were asked if they wanted to receive a transcript post-interview to check for accuracy, none did.

For peer review only

For peer review only - http://bmjopen.bmj.com/site/about/guidelines.xhtml

Table 2. Summary Topic Guides for professional, managerial and support staff, and patient interviews

**Health professionals, managerial and support staff**	**Role in the AKI prevention project**<br>• Current role<br>• How supported patients to prevent AKI before the project?<br>• Preparation for role in sick day guidance/AKI project<br>• Specific training/education<br>• Additional needs for training/education in the area of AKI prevention	**Views of the AKI prevention project**<br>• Who offered sick day rules/other AKI interventions to? (types of patient)<br>• How did you engage with patients<br>• What works well and why? (enablers)<br>• What does not work well and why? (barriers)<br>• Views of its impact on patients<br>• Views of the impact on your work, and the rest of the healthcare team	**Integration with health care**<br>• How do sick day rules/other AKI initiatives, fit/link with other support for AKI prevention?<br>• Fit with long term conditions management and other health needs and services?<br>• How do they fit/link with hospital care/social/voluntary sector?<br>• Contact/interaction with the rest of the primary health care team, secondary care team(s) around sick day guidance/AKI more generally?<br>• Which health care professionals are best placed to provide AKI prevention support?
**Patients**	**Context/history**<br>• Length of time of condition/taking medicines<br>• Perceptions of health and illness in everyday life<br>• Management of medicines and/or acute episodes of illness before the project (whether used a sick day guidance before/blister packs)<br>• Difficulties experienced around managing medicines and any needs?	**The sick day guidance/other kidney health interventions**<br>• How they found out about the service?<br>• Whether used the card or not?<br>• What do they find useful or like about it?<br>• What do they not find useful or dislike about it?<br>• Do they feel it has helped them? If so, how?<br>• Could it be improved? If so, how?<br>• Which healthcare practitioners could/should provide the cards? (where and when)<br>• Who are sick day cards/other AKI interventions suitable for?	**Coordination of care**<br>• Who is involved in their care?<br>• How/where does the sick day guidance/other support provided as part of the project, fit with other services or care received or other self-care undertaken?

For peer review only

Data Analysis

A-M M developed a thematic analysis framework using the evaluation objectives and the four core constructs of Normalisation Process Theory (NPT) to understand implementation.[1516] NPT is concerned with social action rather than attitudes, its four core constructs are coherence (sense-making), cognitive participation (relational work), collective action (operational work) and reflexive monitoring (appraisal). [1516] The NPT constructs provided a pragmatic structure to consider different types of work surrounding the implementation of sick day guidance cards. Furthermore, it provided a sensitising framework to explore the relationships between different types of work being undertaken. [18]The questions asked of the health practitioner interview data included:

- how do they make sense of implementing the sick day card initiative? (coherence)
- what work have they done to implement the initiative? (operational work)
- how is the initiative being communicated or enacted by local others? (relational work)
- what judgments have been made about the initiative? (appraisal)

The questions we asked of the patient participant data included:

- how does the participant make sense of health and illness? (coherence)
- what was the context of the participant receiving a card and guidance?
- how did they make sense of the card and implement the guidance in their day to day lives? (coherence, operational, relational work)
- how did they value the intervention? (appraisal).

As the interviews were completed and transcribed, data from each account was grouped according to role, which resulted in six data sets: GP; practice nurse and health care

For peer review only

assistant; administration; community pharmacist; practice pharmacist; and patient-participant. Thematic analysis using the transcripts, the audio recordings and the field notes was carried out by A-M M and TB within and then across the six data sets to explore similarities and differences in context, sense-making, implementation and appraisal of the card.[19] Key themes and tensions underpinning implementation emerged through comparative analysis of individual and collective working practises underpinning introduction of sick day guidance cards.

Results

A version of the findings of this paper is included in a wider report that has been provided to the funding organisation.[20] Acute Kidney Injury (AKI) was viewed as a new phenomenon and the implementation of sick day guidance cards entailed a new set of working practises. Analysis indicated that AKI prevention guidance was not necessarily a straightforward concept to understand, or to communicate. Health practitioners thought the cards required some knowledge of illness symptoms and medicines, and that patients had to decide how severe the symptoms were before acting, or re-starting their medication. One practice pharmacist stated '*…patients don't understand what fever is…they think that if they've got a headache it's fever…we're trying to explain and they don't understand, or they say well, if I had a bout of diarrhoea do I stop the medication…it's severe. Well, what is severe, you know? Obviously it's very subjective…*' (SKHIP13PP).

Comparative analysis highlighted a tension between the need to achieve reach to the populations deemed at risk (i.e. those taking medicines specified on the card) and at the

For peer review only - http://bmjopen.bmj.com/site/about/guidelines.xhtml

same time ensure comprehension concerning use of the guidance. There was evidence that this tension influenced the implementation of the sick day guidance intervention. The following sections describe the different approaches employed.

Administration of the sick day guidance card in conjunction with face-to-face communication

A common theme was health professionals and patients valuing the need to explain the guidance in person. One patient reflected *'I don't think that it should be just put on a counter… I don't think, number one, they'll read it, number two, they'll digest what's on it, or number three, they'll apply it to themselves'* (SKHIP22PA). A practice nurse thought dialogue was also important to reduce miscommunication, avoid patient confusion and additional GP workload.

'I always explain …There's no point giving someone a card if they don't understand what it's for…my grandma wouldn't understand that. She'd probably misinterpret that and…stop taking everything' (SKHIP25PN).

Analysis of health practitioner and patient accounts revealed that patients responded to the guidance in a variety of ways, not always as intended. One patient participant used the terms sickness and illness interchangeably and spoke of different classifications of illness. She asked which type the guidance card was referring to, to be confident of following the instructions properly.

For peer review only - http://bmjopen.bmj.com/site/about/guidelines.xhtml

‘*What do you define as illness…? Well, I suppose I don't know… I've got arthritis, that's not an illness it's just a thing of life when you get older... I've had spinal surgery, but they're not illnesses*…’ (SKHIP22PA).

Two health practitioners reported instances of patients with medication associated diarrhoea stopping their tablets since receiving a card. This unintended consequence of the initiative, lead to those patients being prescribed alternative medication to alleviate the side effect. A couple of patient participant accounts revealed a lack of willingness to follow the guidance as it had not been implemented by their hospital specialist, whose opinion they trusted, and they did not want to make their condition worse. *‘I’d rather feel sick than have a problem with the high blood pressure…’* (SKHIP31PA).

The concept of temporary cessation of medicines required careful consideration, for example when to stop, restart and what dosage to reinstate. *‘We don’t have enough data or…best practice… if you stop the metformin or whatever medication how long do you stop it for…? Then after a week are you going to restart them again on the ten milligram or are you going to start them on the 1.5, the 2.5…?*’ (SKHIP14GP).

Although valued by the health practitioners interviewed, implementation of sick day guidance initiative demanded extra work. In general practice, this was deemed less problematic when it fitted into existing long-term condition review appointments, particularly with practice nurses or health care assistants. In community pharmacies, implementation sat more readily within face-to-face medication review appointments or opportunistic over-the-counter interactions, including the purchase of non-steroidal anti-

For peer review only - http://bmjopen.bmj.com/site/about/guidelines.xhtml

inflammatory drugs (NSAIDS, such as ibuprofen). One community pharmacist used the purchase of anti-diarrhoeal or sickness medications as an opportunity to administer AKI guidance.

‘*…when people have been coming in to buy stuff for sickness or diarrhoea… If it turns out that they're on one of the medications that’s on the card, then we’ll give them a card then as well and explain about it*’ (SKHIP5CP).

There were limits to the implementation of sick day guidance in patient populations deemed at increased risk of AKI. Concerns were expressed across the health professionals interviewed that the cards and temporary cessation of medications were not suitable for patients with cognitive impairments such as Alzheimer’s disease, reduced literacy in English, those with advanced learning difficulties or visual impairments, or for elderly housebound patients taking multiple medicines. One community pharmacist commented on the difficulties facing patients and carers using dosette box (blister pack) systems.

‘*they* (patients) *might have four or five tiny little white ones, and then if they’re elderly or they can’t see the markings, they don’t know what tablet they should be stopping…. if it was a family member looking out for it, that would be I guess possible, but a lot of the carers are not allowed to alter any medication’* (SKHIP7CP).

Administration of sick day guidance cards to patients in conjunction with telephone consultations

For peer review only - http://bmjopen.bmj.com/site/about/guidelines.xhtml

Phase Two of the project entailed Practice Pharmacists supporting the implementation of the sick day guidance cards in general practices (see Table 1). All of the four CCG employed pharmacists valued and engaged with the project. However, they outlined difficulties fitting the implementation in with their pre-existing workload. There were more patients to work with than anticipated, and the searches, writing to patients, communicating with them and feeding the results back to GPs took longer to complete than the pharmacists described having time for.

To implement the project in this context, a decision was made to have telephone conversations with patients rather than face-to-face interactions. However, this created additional challenges. The phone calls took as long as the face-to-face encounters as the pharmacists expressed a professional need to do things '*properly*'. They reported patients not always being happy to talk with a perceived stranger on the phone about their health. Patient understanding was harder to assess and patients did not necessarily agree to enact the guidance if they became ill. Unlike the face-to-face GP and practice nurse consultations, patients on the other end of the phone had no prior trusting relationship with the practice pharmacist. One pharmacist tried to mitigate some of these issues by talking with a GP in advance of phoning:

'…I'm not going to just pick up the phone and ring this patient now, I'm going to ask the GP what he thinks… for the slightly elderly- some patients, perhaps mental health issues….They obviously know their patients much better than I do so I always take their advice' (SKHIP11PP).

The community pharmacists also spoke of the difficulties of assessing patient comprehension in this way. *'I've had to phone patients …if you've got a query or the*

For peer review only - http://bmjopen.bmj.com/site/about/guidelines.xhtml

prescription will be changed or we'll want to question something …sometimes they're on the ball, they completely know, and sometimes they're just so confused' (SKHIP7CP).

Sick day guidance cards being administered without verbal or written communication

Instructions administered to health practitioners (Figure 2) stressed the need for dialogue with patients to check understanding. However, accounts indicated that this did not always occur. Reasons included other work demands during a practice based consultation, limited time for dialogue, forgetting to discuss it, and some lack of confidence about what to say, partly because of the limited evidence base and so as not to confuse patients, especially those where less fluent in English '*we have quite a lot of different ethnicities here…they've got limited English I think they're not quite sure and it takes quite a while explaining …about what medicines to stop, when to stop it, when to restart it…*' (SKHIP10PN).

Though the community pharmacists were willing to talk with patients about the guidance cards, time shortages and other work demands impinged on implementation. One community pharmacist stated '*Half the time it's remembering to do it because you're thinking about that many different things*' (SKHIP5CP). In addition, they did not always have face-to-face contact with patients '*we've got like 900 of our own patients and we just make the packs and then send them out and delivery, so we don't actually have that much patient contact*' (SKHIP7CP).

Some health practitioners felt that the cards were self-explanatory. One practice nurse said '*vomiting is vomiting and diarrhoea is diarrhoea*' (SKHIP25PN). However, others did not

For peer review only - http://bmjopen.bmj.com/site/about/guidelines.xhtml

agree. One GP thought it was really important to provide patients with written material to aid understanding and compliance '*with certain other sort of medicine regimes, we ask them to stop temporarily if there's a drug interaction and patients are okay with that, as long as you give them sort of written instructions and they know exactly why they're stopping. A lot of it is to do with the understanding. They don't like stopping things if they don't understand why…*' (SKHIP20GP).

A couple of patient accounts referred to finding cards in public information areas of medical practices and community pharmacies. One patient who found a card in this manner wanted to share the sick day guidance message '*…I went into the pharmacy last week, they were on the counter…I picked one up and brought it home …I think it's such a good idea that I've given one to my sister*' (SKHIP22PA).

Communication of AKI risk, but limited use of a sick day guidance card

One GP worked exclusively with patients in care homes across the CCG, which included patients who were diagnosed with cognition limiting conditions such as dementia. Though the guidance messages were deemed pertinent to these groups of patients more vulnerable to AKI, their use was limited due to a potential lack of understanding. *'So we have the card. We didn't use it a lot…We used it to give to the carers. I used it to give to a few of the patients that have capacity'* (SKHIP14GP).

The need for appropriate training for carers, nursing staff and associated social workers was raised, beyond the level of the sick day guidance card. Specifically there was felt to be an on-going need for health practitioners to highlight the importance of fluid management in

For peer review only - http://bmjopen.bmj.com/site/about/guidelines.xhtml

conjunction with medicines management. *'…they* (dementia patients) *ended up not eating or drinking, worsening of the renal function and become unwell and they end up in hospital…' So it's working with the carer as well to understand…. It's serious things that they might die from, not being hydrated'* (SKHIP14GP).

Discussion

Principal Findings

Implementation of sick day guidance cards to prevent community based AKI entailed a new set of working practises. The temporary cessation of medicines during episodes of acute illness was not necessarily a straightforward concept to understand or communicate. Comparative analysis of participants' accounts highlighted a tension between ensuring reach in administration of the cards to at risk populations whilst being confident to ensure patient understanding of their purpose and use.

Strengths and Weaknesses of this study

Unlike an earlier study[21] a key strength of this evaluation was to conduct an in-depth exploration of systematic roll out across a single healthcare setting. The study was hypothesis generating and use of Normalisation Process Theory provided a sensitising framework for data collection and analysis.[15-17] Recognising that all theories have the potential to structure and constrain analysis, NPT was chosen as it ensured that a range of individual and collective working practises were considered during analysis.[1516] Methods to enhance the trustworthiness of the findings, including their transferability, entailed

For peer review only - http://bmjopen.bmj.com/site/about/guidelines.xhtml

exploring types of work undertaken in both general practices and community pharmacies as well as their use by a range of health professionals in these different settings. [22]

The study entailed comparative analysis of both patient and professional accounts in order to explore their use in clinical interactions as well as in everyday life. Theoretical saturation was reached in terms of illuminating the key tension between achieving reach whilst ensuring comprehension. However, further research is required to enhance patient understanding and use. Professional accounts allowed descriptions of experiences of use by patients, though difficulties were encountered recruiting patient participants who had experiences of having used a sick day guidance card at times of acute illness. It is important to acknowledge that only five patients were interviewed in spite of extensive recruitment efforts. Health professionals did not always pass on the evaluation recruitment packs to patients, and the patients we interviewed had not used the cards to date; which could help to explain limited patient involvement. Workload pressures were cited as reasons for health professionals declining to participate in the evaluation.

During the course of the interviews, health practitioners were asked about patient sense-making, use and appraisal of the guidance cards. In light of limited patient involvement these accounts became more important. We acknowledge that they are third order interpretations; our interpretations of what health practitioners reported about patients' sense-making, appraisal and use of the cards. However, the comparative approach taken has facilitated understanding of the pluralistic journeys of the cards and their intended and unintended messages and trajectories from card giver to patient across the 29 interviews. Future studies may benefit from sampling patients who have been coded in general practice as having been provided sick day guidance (i.e. Read Code 8OAG. 'Provision of information

For peer review only - http://bmjopen.bmj.com/site/about/guidelines.xhtml

about Acute Kidney Injury'[23]) and also who have been coded with an episode of acute illness (e.g. gastroenteritis, acute respiratory infection). In doing so, this this would enable purposeful sampling according to medical history including evidence of multi-morbidity. As stated in the CCG report, 106,000 cards (see Table 1) were distributed across general practices and community pharmacies within the time frame of the project. [20] However, community pharmacists were not required to record administration to patients and inaccuracies in coding in general practice limited the potential for a robust quantitative analysis. Future study design would benefit from greater alignment between quantitative and qualitative elements of an evaluation. [20]

Comparison with other studies

In terms of professional responsibility, there are recognised boundaries to the role of general practitioners in supporting self-management.[24] The findings of this study resonate and build on the results of previous research, which highlighted issues around the consistency of clinical message, and the additional work required to reduce the risk of harm from AKI using medicines management interventions.[2125] The intervention was conducted at a time when concern was raised that UK general practice workload may be at 'saturation point.'[26] Results suggested that this influenced engagement with the CCG led initiative.

Though currently available through the Scottish Patient Safety Programme,[6] the findings from this qualitative study resonate with recently published literature, which highlights a need for a more robust evidence base surrounding both the implementation and effectiveness of sick day guidance cards.[27-29] A recent systematic review showed that 'there is no evidence of the impact of drug cessation interventions on AKI incidence during inter-

For peer review only - http://bmjopen.bmj.com/site/about/guidelines.xhtml

current illness in primary or secondary care.'[2729] In terms of implementation, studies evaluating AKI interventions in secondary care indicate that establishing clinician approval is critical with a need for intervention design to take into account 'how technologies, people and organisations dynamically interact' in order for AKI interventions to become integrated into routine clinical practice. [3031]Interventions that disrupt workflow 'may not be sustainable even if there has been a positive impact on care.'[30]

Results from a population-based cohort study indicate that patient co-morbidities including chronic kidney disease are much more strongly associated with AKI and that treatment with either an ACE Inhibitor or an ARB is only associated with a small increase in AKI risk.[28]That is, younger patients with limited comorbidity (e.g. on ACEI for treatment of hypertension) have a low absolute risk of AKI, whilst patients living with multi-morbidity in whom there may be professional concerns about ensuring effective risk communication, have a much higher risk of AKI. [28]

Implications for clinicians, policy makers and future research

In the UK, NICE recommends raising awareness of AKI in higher risk population groups with specific reference to patients who: have existing CKD; have had a previous episode of illness complicated by AKI; and/or have neurological or cognitive impairment and who may be reliant on carers for support with fluid intake during an acute illness (e.g. those with cognitive impairment).[32] This may help address a knowledge gap in patient and public understanding of the importance in the maintenance of kidney health. A survey conducted in 2014 on behalf of NHS England indicated that 'about half of the population in Great Britain don't think their kidneys make urine' and 'only an eighth (12%) of interviewees

For peer review only - http://bmjopen.bmj.com/site/about/guidelines.xhtml

thought their kidneys had a role in processing medicines.'[33] However, the findings from this study suggest an evidence base is urgently warranted to determine how best to resource effective self-management support for higher risk patient populations. Targeting patients who have had an episode of illness complicated by AKI may be particularly important. As a marker of vulnerability, data from a Welsh study showed that around 50% of their patient population died within 14 months; the study also revealed high rates of hospital readmission.[34] Of the 733 patients discharged following a hospital admission complicated by AKI, there were 498 rehospitalisation events in a six month period.[34]

The NHS England Urgent and Emergency Care Review also emphasised the need for better support for people to self-care.[35] Our analysis in conjunction with the research by Mansfield et al (2016),[28] suggest sick day guidance cards alone, that focus solely on temporary cessation of medicines, are unlikely to be sufficient to reduce the harm associated with AKI. The CCG chose to implement the Scottish (NHS Highland) Medicine Sick Day Rules card without significant modification of content or format.[6] However, the current intervention may need modifying, to make it suitable for use with various populations, such as provision in languages other than English. For example, recognising the risks of the 'triple whammy' combination of NSAIDS prescribed in conjunction with diuretics and renin–angiotensin system inhibitors (i.e. ACE inhibitors and ARBs), is there potential for misunderstanding if NSAIDS are included in a sick day guidance card administered to patients with heart failure?[36] Both usability testing as well as experience based co-design are methodological approaches that may optimise the development of an intervention that takes into account patient and carer experience.[37] The findings suggest other strategies may need to be resourced to prevent AKI in people with complex health and social care needs such as those living with dementia. A key issue raised was to provide better education and support for

For peer review only - http://bmjopen.bmj.com/site/about/guidelines.xhtml

carers (both professional and informal). The Royal College of General Practitioners has provided guidance on the development of ‘carer friendly’ practises and the establishment of Patient Participation Groups, may be a mechanism to resource and integrate support for carers into the organisation of acute care.[3839]

Conclusions

The findings from this qualitative evaluation suggest that there are boundaries to the implementation of sick day guidance cards to prevent Acute Kidney Injury in primary care. A common theme was the need to ensure patient understanding of their purpose and use. Communicating the concept of temporary cessation of medicines was a particular challenge and limited their administration to patient populations at higher risk of AKI, particularly those with less capacity to self-manage. The analysis suggests that sick day guidance cards that focus solely on medicines management may be of limited benefit without either adequate resourcing, or if delivered as a standalone intervention. Development and evaluation of a primary care intervention encompassing a range of initiatives to tackle the harm associated with AKI is warranted.

For peer review only - http://bmjopen.bmj.com/site/about/guidelines.xhtml

Declarations

Ethics approval and consent to participate

Ethical approval was gained from Leeds West Research Ethics Committee (REC Reference Number: 15/YH/0174). Informed consent was gained from all participants prior to interview.

Consent for publication

Not applicable.

Availability of data and material

The data has been stored securely with password protected files to ensure confidentiality, in keeping with the research protocol and good data management guidelines. It will not be shared.

Competing interests

The authors declare that they have no competing interests.

Funding

This project was funded by the National Institute for Health Research Collaboration for Leadership in Applied Health Research and Care (NIHR CLAHRC) Greater Manchester and NHS Salford CCG. The NIHR CLAHRC Greater Manchester is a partnership between providers and commissioners from the NHS, industry and the third sector, as well as clinical and research staff from the University of Manchester. The views expressed in this article are those of the authors and not necessarily those of the NHS, NIHR or the Department of Health".

For peer review only - http://bmjopen.bmj.com/site/about/guidelines.xhtml

Authors Contributions

TB, RE, SS, SM and SH conceived and designed the study, A-MM and RE collected the data, TB and A-MM analysed the data. A-M M, RE and TB all have extensive experience of designing, conducting and analysing qualitative health research at doctoral and post-doctoral level. All authors contributed to writing the manuscript and have read and approved the final manuscript.

Acknowledgements

The authors would like to thank the project steering group for their input and guidance throughout the study.

Authors' information (optional)

TB is a member of NHS England's Think Kidneys Programme Board.

For peer review only

For peer review only - http://bmjopen.bmj.com/site/about/guidelines.xhtml

References

1. Mehta RL, Cerdá J, Burdmann EA, et al. International Society of Nephrology's 0by25 initiative for acute kidney injury (zero preventable deaths by 2025): a human rights case for nephrology. *The Lancet*;385(9987):2616-43.

2. Kidney Disease Improving Global Outcomes (KDIGO) Acute Kidney Injury Work Group. KDIGO Clinical Practice Guideline for Acute Kidney Injury. *Kidney International Supplement* 2012;2(1):1–138.

3. NHS England, UK Renal Registry, Think Kidneys. Acute Kidney Injury Best Practice Guidance: Responding to AKI Warning Stage Test Results for Adults in Primary Care: Think Kidneys, 2016.

4. National Institute for Health and Care Excellence. Acute Kidney Injury: prevention, detection and management (CG169): NICE, 2013.

5. NHS England, UK Renal Registry, Think Kidneys. Acute Kidney Injury Warning Alert Best Practice document: Think Kidneys, 2014.

6. NHS Scotland, Health Improvement Scotland, Scottish Patient Safety Programme. Medicine Sick Day Rules Cards [internet]. NHS Scotland, Health Improvement Scotland; 2016 Availablefrom:http://www.scottishpatientsafetyprogramme.scot.nhs.uk/programmes/primary-care/medicine-sick-day-rules-card [accessed 4 April, 2017].

7. Kerr M, Bedford M, Matthews B, et al. The economic impact of acute kidney injury in England. *Nephrology Dialysis Transplantation* 2014;29(7):1362-68.

8. Morrison C, Wilson, M. Medicine Sick Day Rules Cards Intermin Evaluation. [internet] NHS Highland. July 2014 Available from http://www.knowledge.scot.nhs.uk/media/CLT/ResourceUploads/4055542/NHSH%20interim%20evaluation%20medicine%20sick%20day%20rules.pdf [accessed 7 January 2015].

9. Feehally J, Gilmore I, Barasi S, et al. RCPE UK consensus conference statement: management of acute kidney injury: the role of fluids, e-alerts and biomarkers. *J R Coll Physicians Edinb* 2013;43 doi: 10.4997/jrcpe.2013.109.

For peer review only - http://bmjopen.bmj.com/site/about/guidelines.xhtml

10. Scottish Government. NHS Scotland Polypharmacy guidance [internet]. March 2015. Available from: http://www.sehd.scot.nhs.uk/publications/DC20150415polypharmacy.pdf [accessed 2 April 2015].

11. Morrison C, Wilson M. Medicine Sick Day Rules cards: a safe and effective tool to improve medicines safety in NHS Highland [internet]. *International Journal of Pharmacy* Practice 2015;23;S2. DOI: 10.1111/ijpp.12213. Available fromhttp://onlinelibrary.wiley.com/doi/10.1111/ijpp.12213/pdf.

12. NHS England, UK Renal Registry. [internet] Think Kidneys Available from http://www.thinkkidneys.nhs.uk/ accessed 7 Apr 2015.

13. NHS England, UK Renal Registry, Think Kidneys. "Sick day" guidance in patients at risk of Acute Kidney Injury: an Interim Position Statement from the Think Kidneys Board: Think Kidneys, 2015.

14. Hoffmann TC, Glasziou PP, Boutron I, et al. Better reporting of interventions: template for intervention description and replication (TIDieR) checklist and guide. *BMJ* 2014;348 doi: 10.1136/bmj.g1687.

15. May C, Mair F, Finch T, et al. Development of a theory of implementation and integration: normalisation process theory. *Implement Sci* 2009;4.

16. Murray E, Treweek S, Pope C, et al. Normalisation process theory: a framework for developing, evaluating and implementing complex interventions. *BMC Med* 2010;8:63.

17. normalizationprocess.org [internet]. Normalization Process Theory On-line Users' Manual, Toolkit and NoMAD instrument; 201.5 Available from http://www.normalizationprocess.org [accessed 10 October 2015].

18. Jung JY, Blakeman T, Hegarty J, Humphreys J, Harvey G. Understanding the implementation of interventions to improve the management of chronic kidney disease in primary care: a rapid realist review. *Implementation Science* 2016;11:47. DOI: 10.1186/s13012-016-0413-7.

19. Silverman S. Interpreting Qualitative Data: Methods for Analysing Talk, Text and Interaction. London: Thousand Oaks 2001.

20. NIHR Collaboration for Leadership in Applied Health Research and Care Greater Manchester. Salford Clinical Commissioning Group Sick Day Guidance Project: Final Report. Manchester: NIHR CLAHRC GM, 2016.

For peer review only - http://bmjopen.bmj.com/site/about/guidelines.xhtml

21. Morris RK AD, Phipps D, Bower P et al. Preventing Acute Kidney Injury: A qualitative study exploring 'sick day rules' implementation in primary care. *BMC Family Practice* 2016;FAMP-D-15-00010R3 (Accepted for Publication 12 July 2016)

22. Lincoln Y, Guba EG. Naturalistic Inquiry. London: Sage; 1985.

23. NHS North West Commissiong Support Unit. Salford Standards Read Code Dictionary. Specification for Read V2 coding for: Salford Clinical Commissioning Group: NHS North West Commissioning Support Unit, 2016.

24. Blakeman T, Macdonald W, Bower P, et al. A qualitative study of GPs' attitudes to self-management of chronic disease. *Br J Gen Pract* 2006;56(527):407-14.

25. Phipps DL, Morris RL, Blakeman T, et al. What is involved in medicines management across care boundaries? A qualitative study of healthcare practitioners' experiences in the case of acute kidney injury. *BMJ Open* 2017;7(1) doi: 10.1136/bmjopen-2016-011765

26. Hobbs FDR, Bankhead C, Mukhtar T, et al. Clinical workload in UK primary care: a retrospective analysis of 100 million consultations in England, 2007-14. *The Lancet* 2016; 287 (10035): 2323-2330.

27. Morden A, Horwood J, Whiting P, et al. The risks and benefits of patients temporarily discontinuing medications in the event of an intercurrent illness: a systematic review protocol. *Systematic Reviews* 2015;4(1):1-6. doi: 10.1186/s13643-015-0135-y

28. Mansfield KE, Nitsch D, Smeeth L, et al. Prescription of renin–angiotensin system blockers and risk of acute kidney injury: a population-based cohort study. *BMJ Open* 2016;6(12) doi: 10.1136/bmjopen-2016-012690

29. Whiting P MA, Tomlinson LA, Caskey F, et al. What are the risks and benefits of temporarily discontinuing medications to prevent acute kidney injury? A Systematic Review and meta-analysis. *BMJOpen* 2017;bmjopen-2016-012674.R1

30. Kanagasundaram NS, Bevan MT, Sims AJ, Heed A, Price DA, Sheerin, NS. Computerized clinical decision support for the early recognition and management of acute kidney injury: a qualitative evaluation of end-user experience. *Clin Kidney J* (2016) 9 (1): 57-62. DOI: https://doi.org/10.1093/ckj/sfv130.

For peer review only - http://bmjopen.bmj.com/site/about/guidelines.xhtml

31. Oh J, Bia JR, Ubaid-Ullah M, Testani JM, Wilson FP. Provider acceptance of an automated electronic alert for acute kidney injury. *Clin Kidney J* (2016) 9 (4): 567-571. DOI: https://doi.org/10.1093/ckj/sfw054.

32. National Institue for Health and Care Excellence. Acute kidney injury Quality standardnice.org.uk/guidance/qs76: NICE, 2014.

33. NHS England, UK Renal Registry, Think Kidneys. Understanding what the public know about their kidneys and what they do. Findings from Ipsos MORI survey – July 2014: Think Kidneys, 2014.

34.Wonnacott A, Meran S, Amphlett B, Talabani B, Phillips A. Epidemiology and outcomes in community-acquired versus hospital-acquired AKI. *Clin J Am Soc Nephrol*. 2014 Jun 6;9(6):1007-14. doi: 10.2215/CJN.07920713.

35. NHS England. High quality care for all, now and for future generations: Transforming urgent and emergency care services in England, Urgent and Emergency Care Review. Leeds: Urgent and Emergency Care Review Team, NHS England, 2013.

36. Dreischulte T, Morales DR, Bell S, Guthrie B. Combined use of nonsteroidal anti-inflammatory drugs with diuretics and/or renin-angiotensin system inhibitors in the community increases the risk of acute kidney injury. *Kidney Int*. 2015 Aug;88(2):396-403. doi: 10.1038/ki.2015.101

37. Locock L, Robert G, Boaz A, et al. Testing accelerated experience-based co-design: a qualitative study of using a national archive of patient experience narrative interviews to promote rapid patient-centred service improvement. Southampton (UK): NIHR Journals Library; 2014 Feb. (*Health Services and Delivery Research*, No. 2.4.) Available from: https://www.ncbi.nlm.nih.gov/books/NBK259580/ doi: 10.3310/hsdr02040

38. Royal College of General Practitioners. RCGP Supporting Carers in General Practice: Summary report on GP practice journeys towards improved carer identification and support. London: Royal College of General Practitioners, 2013.

39. NHS Employers. 2015/16 General Medical Services (GMS) contract: Guidance for GMS contract 2015/16: NHS Employers, 2015.

For peer review only - http://bmjopen.bmj.com/site/about/guidelines.xhtml

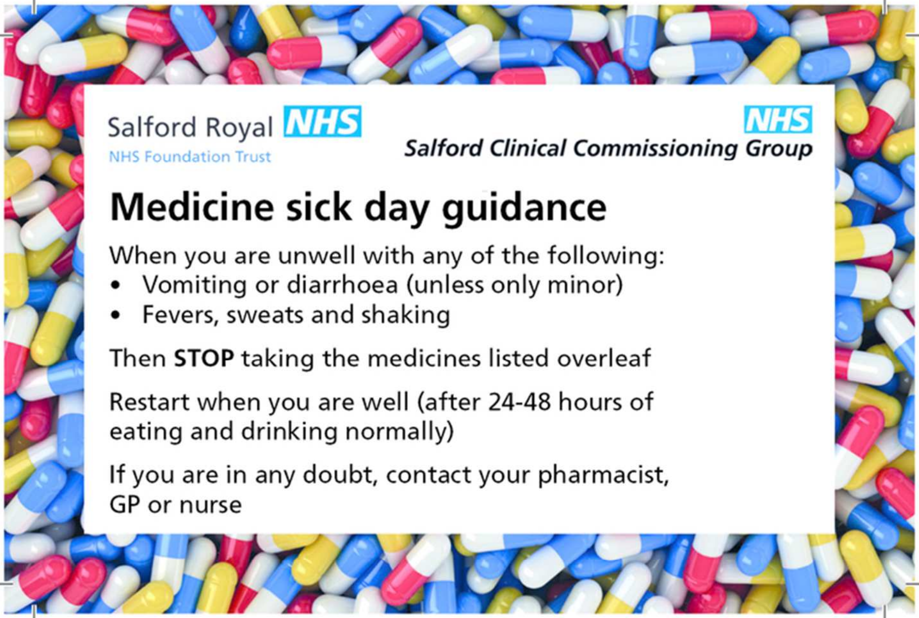

Figure 1 Sick day guidance card used during this project

69x48mm (300 x 300 DPI)

For peer review only - http://bmjopen.bmj.com/site/about/guidelines.xhtml

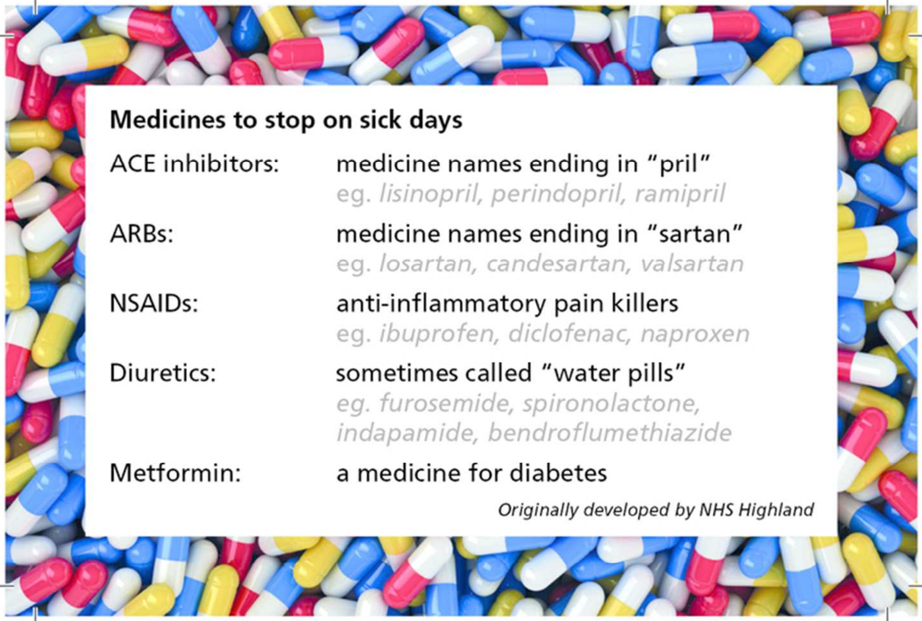

Figure 1 Sick day guidance card used during this project

69x49mm (300 x 300 DPI)

view only

MEDICINES AND DEHYDRATION: SICK DAY GUIDANCE

Offer the following information at the time of giving the card

- Some medicines shouldn't be taken when you have an illness that makes you dehydrated. This is because they can either increase the risk of dehydration or because dehydration can lead to potentially serious side effects of the medicine.
- The medicine you are taking that falls into this category is [tell patient which medicine].
- Illnesses that can cause dehydration are vomiting, diarrhoea and fever.
- This advice does not apply to minor sickness or diarrhoea, which means a single episode.
- If your medicines are in a blister pack you must take it to the chemists so the chemist can show you which ones you need to stop.
- If you have heart failure you may stop these medicines for a maximum of 48 hours but after that you need to contact your GP or heart failure team for further advice.

The list of medicines on the card is not exhaustive but they are highlighted because:

- diuretics can cause dehydration or make dehydration more likely in an ill patient;
- ACE inhibitors, angiotensin II receptor blockers and NSAIDs may impair kidney function in a dehydrated patient, which could lead to kidney failure;
- metformin dehydration increases the risk of lactic acidosis, a serious and potentially life-threatening side effect of metformin.

Figure 2 Guidance provided to health practitioners (shortened form)

only

Understanding the implementation of 'sick day guidance' to prevent Acute Kidney Injury across a primary care setting in England: a qualitative evaluation

COREQ Table

Guide question		Response	Manuscript page number
1	Interviewer/facilitator	The interviews were conducted by AM-M and RE.	13
2	Credentials	AM-M Ba, Ma, PhD<br>RE Ba, Ma, PhD<br>SH BSc, PhD<br>SM MBChB<br>SS MBChB MRCP(UK) PhD<br>TB MBChB; PhD	1-2
3	Occupation	A-M M Post-doctoral research associate, Collaboration for Leadership in Applied Health Research and Care Greater Manchester) (CLAHRC GM),<br>RE Research Fellow, CLAHRC GM,<br>SH Kidney Research Programme Manager, CLAHRC GM<br>SM Local clinical research speciality lead and GP, NHS Salford Clinical Commissioning Group<br>SS Senior renal consultant, Salford Royal NHS Foundation Trust<br>TB Clinical Senior Lecturer and GP, CLAHRC GM.	1-2
4	Gender	A-M M female<br>RE female<br>SH female<br>SM female<br>SS female<br>TB male	1-2
5	Experience and training	A-M M is a trained social anthropologist and experienced health researcher<br><br>RE is an experienced and trained qualitative researcher in health<br><br>TB is an experienced and trained qualitative health researcher<br><br>SH is an experienced post-doctoral health research manager<br><br>SS and SM are both health professionals with experience of designing kidney health research and interventions.	31<br><br>31<br><br>31<br><br>Tidier Table page 8, item 5 'Who'<br><br>Tidier Table page 8, item 1 'Why', item 5 'Who'.

For peer review only

Guide question		Response	Manuscript page number
6	Relationship established	The researchers conducting the interviews (A-M M, RE) did not know any of the interviewees prior to the research.	13
7	Participant knowledge of the researcher	Via information sheets and verbal clarification the participants knew that A-M M and RE were researchers evaluating the acute kidney injury (AKI) sick day guidance initiative.	13
8	Researcher characteristics	The researchers were working on a (CLAHRC) programme of research on kidney ill-health prevention in Greater Manchester.	Page 8, Tidier Table, item 1, 'Why'
9	Methodological orientation and theory	We used normalisation process theory (NPT) to inform the design in the interview topic guide and analysis, NPT aims to explore the contexts, sense-making, activity and participant appraisal of an implementation project.	Page 12, page 15 Table 2, and pages 16-17.
10	Sampling	Each pharmacy and GP practice in the locality was asked to take part in the evaluation and to help recruit patient participants via dissemination of information packs. We asked those we interviewed if they knew others who might want to take part.	12
11	Method of approach	Participants were contacted by email and post.	12 and 13
12	Sample size	29 participants.	12, 13
13	Non-participation	'Workload pressures were cited as reasons for health professionals declining to participate in the evaluation.'	25
14	Setting of data collection	Data were collected at the participants' place of work and over the phone.	13
15	Presence of non-participants	Not applicable.	
16	Description of sample	29 participants comprised of: seven GPs; five practice nurses; five community pharmacists; four practice based pharmacists; two managers (one medical practice manager and one community pharmacy manager); and a health care assistant.	12
17	Interview guide	See Table 2. Summary Topic Guides for professional, managerial and support staff, and patient interviews.	15
18	Repeat interviews	Not applicable.	
19	Audio/visual recording	Audio recorded, with consent from the participant, as explained in verbal and written information.	13
20	Field notes	Written after each interview and used to inform analysis.	13, 17
21	Duration	From 9 minutes to 66 minutes (median = 33 minutes).	13
22	Data saturation	Participants were recruited until saturation of key emergent theme. Practical consideration also determined end point in recruitment.	25
23	Transcripts returned	Participants were asked if they wanted a copy of the transcript, none did.	14
24	Number of data coders	2, data analysis was conducted by A-M M and TB.	17
25	Description of the	Analysis was focused around NPT and the evaluation	16-17

For peer review only

Guide question		Response	Manuscript page number
	coding tree	objectives.	
26	Derivation of themes	Analysis was focused around the four constructs of NPT coherence (sense-making), cognitive participation (relational work), collective action (operational work) and reflexive monitoring (appraisal); the evaluation objectives and we allowed for serendipitous findings. Sense-making, activities and evaluation of card comprehension and use emerged through engagement with these constructs.	16-24
27	Software	Ms Word (transcripts, and fieldnotes) and an audio recorder were utilised to record and help make sense of the data.	17
28	Participant checking	Participants were not asked to provide feedback on the findings.	
29	Quotations presented	Quotations are presented throughout the results section.	17-24
30	Data and findings consistent	The themes arising stem from the data and are consistent throughout.	17-24
31	Clarity of major themes	The major themes are presented under headings and under the heading principal findings.	17-24, 24.
32	Clarity of minor themes	Minor themes are incorporated into the themes outlining how sick day guidance cards were implemented in routine practice.	17-24

For peer review only

BMJ Open

Understanding the implementation of 'sick day guidance' to prevent Acute Kidney Injury across a primary care setting in England: a qualitative evaluation

Journal:	*BMJ Open*
Manuscript ID	bmjopen-2017-017241.R2
Article Type:	Research
Date Submitted by the Author:	15-Aug-2017

Complete List of Authors:	Martindale, Anne-Marie; National Institute for Health Research Collaboration for Leadership in Applied Health Research and Care Greater Manchester; The University of Manchester, Centre for Primary Care, 5th Floor, room 5.02, Williamson Building, Oxford Road, M13 9PL. Fax 0161 275 7600<br>Elvey, Rebecca ; National Institute for Health Research Collaboration for Leadership in Applied Health Research and Care Greater Manchester; The University of Manchester, 5th Floor, Suite 6, Williamson Building, Oxford Road, M13 9PL.<br>Howard, Susan; National Institute for Health Research Collaboration for Leadership in Applied Health Research and Care Greater Manchester; Salford Royal NHS Foundation Trust, 3rd Floor, Mayo Building, Stott Lane, M6 8HD.<br>McCorkindale, Sheila; NHS Salford Clinical Commissioning Group, 7th Floor, St James's House Pendleton Way Salford, M6 5FW<br>Sinha, Smeeta; Salford Royal NHS Foundation Trust, Stott Lane, M6 8HD<br>Blakeman, Tom; National Institute for Health Research Collaboration for Leadership in Applied Health Research and Care Greater Manchester; The University of Manchester, 6th Floor, Suite 6, Williamson Building, Oxford Road, M13 9PL

**Primary Subject Heading**:	General practice / Family practice
Secondary Subject Heading:	Communication, Qualitative research
Keywords:	Acute kidney injury, PREVENTIVE MEDICINE, PRIMARY CARE, QUALITATIVE RESEARCH, patient safety

SCHOLARONE™ Manuscripts

Understanding the implementation of 'sick day guidance' to prevent Acute Kidney Injury across a primary care setting in England: a qualitative evaluation

Authors

Anne-Marie Martindale (corresponding author)

National Institute for Health Research Collaboration for Leadership in Applied Health Research and Care, Greater Manchester, UK.

University of Manchester, 5th Floor, room 5.02, Williamson Building, Oxford Road, Manchester, UK, M13 9PL. Fax 0161 275 7600.

Telephone 0161 275 7601

anne-marie.martindale@manchester.ac.uk

Rebecca Elvey

National Institute for Health Research Collaboration for Leadership in Applied Health Research and Care, Greater Manchester, UK.

University of Manchester, 5th Floor, Suite 6, Williamson Building, Oxford Road, Manchester, UK, M13 9PL. Rebecca.elvey@manchester.ac.uk

Susan J Howard

National Institute for Health Research Collaboration for Leadership in Applied Health Research and Care, Greater Manchester, UK.

Salford Royal NHS Foundation Trust, 3rd Floor, Mayo Building, Stott Lane, Salford, UK, M6 8HD. Susan.howard@srft.nhs.uk

Sheila McCorkindale

NHS Salford Clinical Commissioning Group, 7th Floor, St James's House Pendleton Way, Salford, UK, M6 5FW. sheila.mccorkindale@nhs.net

Smeeta Sinha

For peer review only

Salford Royal NHS Foundation Trust, Stott Lane, Salford, UK, M6 8HD.

smeeta.sinha@srft.nhs.uk

Tom Blakeman

National Institute for Health Research Collaboration for Leadership in Applied Health Research and Care, Greater Manchester, UK.

University of Manchester, 6th Floor, Suite 6, Williamson Building, Oxford Road, Manchester, UK, M13 9PL. tom.blakeman@manchester.ac.uk

Keywords

Acute Kidney Injury; Preventative medicine, Primary care; Qualitative research; patient safety

Revised word count 5153 (excluding title page, references, figures and tables)

For peer review only

For peer review only - http://bmjopen.bmj.com/site/about/guidelines.xhtml

Understanding the implementation of 'sick day guidance' to prevent Acute Kidney Injury across a primary care setting in England: a qualitative evaluation.

Abstract

Objectives: The study sought to examine the implementation of sick day guidance cards designed to prevent acute kidney injury (AKI), in primary care settings.

Design: Qualitative semi-structured interviews were conducted and comparative analysis informed by Normalisation Process Theory (NPT) was undertaken to understand sense-making, implementation and appraisal of the cards and associated guidance.

Setting: A single primary care health setting in the North of England.

Participants: 29 participants took part in the qualitative evaluation: 7 GPs, 5 practice nurses, 5 community pharmacists, 4 practice pharmacists, 2 administrators, 1 health care assistant, and 5 patients.

Intervention: The sick day guidance intervention was rolled out (2015-2016) in general practices (n=48) and community pharmacies (n=60). The materials consisted of a 'medicine sick day guidance' card, provided to patients who were taking the listed drugs. The card provided advice about medicines management during episodes of acute illness. An information leaflet was provided to healthcare practitioners and administrators suggesting how to use and give the cards.

For peer review only - http://bmjopen.bmj.com/site/about/guidelines.xhtml

Results: Implementation of sick day guidance cards to prevent AKI entailed a new set of working practises across primary care. A tension existed between ensuring reach in administration of the cards to at risk populations whilst being confident to ensure patient understanding of their purpose and use. Communicating the concept of temporary cessation of medicines was a particular challenge and limited their administration to patient populations at higher risk of AKI, particularly those with less capacity to self-manage.

Conclusions: Sick day guidance cards that focus solely on medicines management may be of limited patient benefit without adequate resourcing, or if delivered as a standalone intervention. Development and evaluation of primary care interventions is urgently warranted to tackle the harm associated with AKI.

284 words

For peer review only - http://bmjopen.bmj.com/site/about/guidelines.xhtml

Strengths and limitations of this study

- Using Normalisation Process Theory (NPT) has allowed important insights to emerge into the comprehension, use and appraisal of the AKI sick day card initiative.
- Interviews with a range of professionals (GPs, nurses, community and practice-based pharmacists, a health care assistant, practice administrators) and patients enhanced understanding of the individual and collective working practises surrounding the professional implementation AKI sick day guidance cards.
- Patient recruitment to the qualitative evaluation via general practice was slow and yielded only five patient-participants. This limited analysis of patient use of sick day guidance in everyday life.
- Future study design would benefit from greater alignment between quantitative and qualitative elements of an evaluation.

For peer review only

For peer review only - http://bmjopen.bmj.com/site/about/guidelines.xhtml

Introduction

Addressing the harm related to Acute Kidney Injury (AKI) is a worldwide priority.[1] AKI is characterised as a sudden reduction in kidney function over hours or days.[2-4] It is a marker of illness severity and is seen as a 'force multiplier,' complicating episodes of acute illness.[3] As a clinical syndrome, the majority of cases of AKI are due to a combination of underlying infection, hypovolaemia (low circulatory blood volume), hypotension (low blood pressure) and medication effects.[3] Addressing these potentially modifiable factors are central to both the prevention and management of AKI and its associated burden.[2-4]

Across the United Kingdom, patient safety initiatives have been established to address the morbidity, mortality and costs linked to AKI.[2 5-7] In Scotland, informed by findings from a primary care study conducted by NHS Highland, medicine sick day rules have been made available nationally through the Scottish Patient Safety Programme.[6 8] The introduction of medicine sick day rules relates to NHS Scotland Polypharmacy Guidance as well as national guidance, published by the National Institute for Health and Care Excellence (NICE) and by the Royal College of Physicians of Edinburgh UK. These publications highlight a need to consider temporary cessation of medicines at times of acute illness.[4 9 10]That is, during these episodes, 'any drug that reduces blood pressure, circulating volume, or renal blood flow' increases the risk of AKI.[3] Medicines that exacerbate this risk include NSAIDS (non-steroidal anti-inflammatory drugs), diuretics, ACE inhibitors and angiotensin II receptor blockers (ARBs).[3] In addition, the Scottish medicine sick day rules refer to the temporary cessation of metformin, which may accumulate at times of reduced kidney function, resulting in an increased risk of adverse effects.[6] The NHS Scotland 'Medicine Sick Day Rules' cards were

For peer review only - http://bmjopen.bmj.com/site/about/guidelines.xhtml

developed through extraction of NHS Scotland Polypharmacy Guidance (2012) and were 'designed with input from pharmacists, doctors and patients.' [10 11] They provide instructions on temporarily stopping these specific types of medicines during episodes of acute illness.[6 8]

In England, within NHS England's Patient Safety Domain, the Think Kidneys Programme (https://www.thinkkidneys.nhs.uk) was established to tackle the harm associated with AKI.[12] Through the programme, resources have been developed for primary and secondary care, including an Interim Position Statement on 'Sick Day' Guidance, which highlights a clinical equipoise surrounding the systematic implementation of sick day guidance. [13]

It was in this wider context that a Clinical Commissioning Group (CCG), in partnership with the local hospital, embarked on service improvement initiatives to address the harm associated with AKI. Informed directly by the Scottish approach in conjunction with national guidance[4 6 8] the CCG sought to implement the use of sick day guidance across general practices and community pharmacies within its boundaries. The Sick Day Guidance Project including an overview of the organisation of primary health care in England is outlined in Table 1 as well as Figures 1, 2 and 3. In accordance with NHS England Think Kidneys guidance, the project entailed formal evaluation. With a view to provide a platform for future larger scale evaluation, the study sought to explore and understand processes underpinning the implementation of sick day guidance in primary care.

For peer review only - http://bmjopen.bmj.com/site/about/guidelines.xhtml

Table 1 The Sick Day Guidance Project TIDIER[14]

TIDieR Item	Brief Description
**Name**	Salford Kidney Implementation Project
**1 Why**	The Salford Partnership for Advancing Renal Care (SPARC) was established to ensure a shared strategy and optimise kidney care across the City.<br>The ambition of sick day guidance is to reduce the risk of avoidable harm to patients taking certain medications. Salford CCG in collaboration with SPARC defined the original implementation design of the sick day guidance intervention.<br>NIHR Collaboration for Leadership in Applied Health Research and Care (CLAHRC) Greater Manchester works in partnership with Salford CCG to support implementation and evaluation of projects. NIHR CLAHRC Greater Manchester evaluated this CCG priority and supported the implementation of sick day guidance.
**2 What**	Medicines sick day guidance in two phases of work.
**3 Materials**	• Sick day guidance cards that suggested the temporary cessation of medicines during bouts of sickness were produced, the text was replicated from the NHS Highland sick day rules card.<br>• Two, one and a half hour, educational events were run for healthcare professionals, organised and delivered by the Steering Group. This included why AKI is important from a local and national context.<br>• Information leaflet outlining the sick day guidance project and guidance on how to use the sick day guidance cards, and poster summarising this information for use in practice.<br>• Poster for patients promoting the sick day guidance card intervention to be used in waiting areas.
**4 Procedures**	1. Training was offered to all general practitioners, practice nurses and the wider practice team, and to community pharmacists for the sick day guidance card implementation.<br>2. During Phase One, the cards were distributed to all community pharmacies and general practices accompanied by an information leaflet and poster with patient engagement instructions. Distribution was carried out by project facilitators face to face, to explain and address any questions arising.<br>3. Two further face to face visits were made to each general practice and pharmacy by the NIHR CLAHRC GM project team to reinforce the project/provide additional materials/support.<br>4. The cards were to be provided to patients receiving the drugs listed on the card by general practices and community pharmacies.<br>5. Posters were displayed in practice waiting areas promoting the intervention to patients<br>6. General Practitioners and other practice staff were advised to record the intervention in Salford Integrated Records using Read code 80AG.<br>7. During Phase Two, the practice-based pharmacists accessed patient health records from Salford Royal NHS Foundation Trust to identify those at risk of AKI and constructed a database to record relevant data.<br>8. The practice-based pharmacists were to contact and educate patients on the sick day guidance and to issue a card. They were also expected to complete a medications review.<br>9. Approval was sought to ensure the project was in keeping with national Think Kidneys guidance.
**5 Who**	• The NIHR CLAHRC GM project team, (facilitation, project management, and research staff).<br>• The Steering Group (clinical, pharmacist and managerial staff at Salford CCG and Salford Royal NHS Foundation Trust, plus the NIHR CLAHRC GM project team).<br>• Salford CCG general practices and community pharmacies.
**6 How**	The initial recruitment of general practitioners onto the project was implemented via email, and then three face to face visits were delivered per practice/pharmacy by NIHR CLAHRC GM project team to ensure full understanding of the sick day guidance project. Support was also gained from the local pharmaceutical committee.
**7 Where**	General practices [48] and community pharmacies [60] in Salford. 106,000 cards were

For peer review only - http://bmjopen.bmj.com/site/about/guidelines.xhtml

	provided to general practices and community pharmacies for administration to patients.<br><br>In England, there were structural changes to the health service in 2013 and clinical commissioning groups (CCGs) were formed. Each CCG covers the population of a defined area (that is, patients registered at general practices within the area) and is responsible for planning and commissioning the majority of health services in that area. Primary health care services are provided by general practitioners (GPs) community pharmacies, dentists and opticians. Patients register with a GP practice and attend that practice for appointments with a GP(s). Community pharmacies, also known as local chemist shops, are found on most local high streets, in shopping centres and also in many large supermarkets. Community pharmacies dispense prescription medicines, sell other (non-prescription) medicines and various other goods (typically health-related, baby and cosmetic products) and also provide other services, such as medicines use reviews (MURs). Patients do not register with a community pharmacy and may use any pharmacy (for dispensing or other services), although many patients become regular users of their local pharmacy. Pharmacists also work in general practices; such 'practice-based' pharmacists review medicines prescribing and take part in projects, such as the 'sick day guidance' intervention described here.
**8 When and how much**	Cards were to be provided to a patient, when they attended a general practice appointment or visited a pharmacy between March 2015 and January 2016.<br>Practice pharmacists contacted patients who fit within their criteria for being at risk of AKI.
**9 Tailoring**	Whilst guidance on the explanation to give patients (described above) was provided, professionals were expected to use their professional judgement in deciding how to deliver the intervention.
**10 Modifications**	• Opportunistic observations were conducted during facilitation visits.<br>• Cards were noticed on pharmacy counters, which were available for anyone visiting the pharmacy to pick up and take.<br>• Practice pharmacists encountered difficulties around the process of completing the record searches and communicating with patients in that there was not enough time to do this, consequently, no face to face appointments took place and pharmacists tried to contact patients by telephone.<br>• One practice pharmacist developed their own AKI patient information sheet that was posted out with cards.
**11 How well (planned)**	Adherence and fidelity were not formally assessed, however, the facilitation visits were designed to provide flexible, on-going support and advice on delivering the intervention, and an understanding of how well the intervention was operating in practice was gained through these visits.
**12 How well (actual)**	Practice pharmacists encountered barriers to obtaining the information they needed.<br>• (CLAHRC facilitators gained understanding through their visits and the qualitative evaluation formally researched experiences of implementation – both these are documented in the CCG report).<br>• Sustained efforts had to be made to recruit health professionals and patients via medical practices.

INSERT FIGURES 1 AND 2

INSERT FIGURE 3

For peer review only - http://bmjopen.bmj.com/site/about/guidelines.xhtml

Methods

Study Design

Aligned with the project objectives, Normalisation Process Theory (NPT) provided a sensitising framework to inform the topic guide and explore the context, administration, interpretation and use of sick day guidance cards across a single primary healthcare setting in England. [15,16]NPT is a theory of implementation developed through an in-depth analysis of chronic illness care in general practice. [15]It is a sociological theory that provides a structure to explore the individual and group work that people do surrounding the implementation of a complex intervention.[15,16,17]

Data Sampling

To explore the trajectory of implementation across the CCG, all general practices (n = 48), community pharmacies (n = 60) and practice based pharmacists (n = 4) involved in the project were invited to take part in the evaluation. Information packs were provided to explain what involvement entailed. To facilitate patient participant engagement, general practices and community pharmacists were asked to provide information packs to patients who had received a card via a health practitioner. The final data sample of 29 interviews comprised: seven GPs; five practice nurses; five community pharmacists; four practice based pharmacists; two managers (one medical practice manager and one community pharmacy manager); and a health care assistant, a person qualified to carry out routine health care tasks.

Data Collection

For peer review only - http://bmjopen.bmj.com/site/about/guidelines.xhtml

Two qualitative researchers (AM-M; RE) conducted the 29 semi-structured interviews. These were conducted with participants across the CCG between June 2015 and April 2016. Participants received an approved participant information sheet and consent form via post or email. Both were read by the researcher prior to interview and participants had the opportunity to ask questions, and have them answered satisfactorily. Informed consent was gained before each interview. Interviews with the GPs, practice nurses, administrators and the health care assistant took place in private locations within their general practices. Interviews with community pharmacists were also held at private locations at their places of work. Interviews with patients occurred at their homes. Interviews with three of the practice-based pharmacists took place at their place of work; one took place on the phone. The two researchers did not know any of the participants prior to interview. The interviews ranged in length from 9 minutes to 66 minutes (median = 33 minutes). They were digitally audio-recorded in compliance with participants' consent and professionally transcribed.

Interview topic guides were developed to explore the work being undertaken by professionals and patients surrounding the use of sick day guidance cards. NPT was used to inform the areas of questioning.[16] Topics for the health practitioners included previous knowledge of AKI and involvement in kidney health initiatives, their role in the intervention, sense-making, and experiences of implementing and appraising the administration of sick day guidance cards. For patient-participant interviews, topics included: sense-making around health and illness; the context of card giving and guidance explanation; and comprehension and use of the guidance (Table 2). Field notes about the encounter were written immediately after leaving the interview site and used to inform the analysis.

For peer review only - http://bmjopen.bmj.com/site/about/guidelines.xhtml

Participants were asked if they wanted to receive a transcript post-interview to check for accuracy, none did.

For peer review only

For peer review only - http://bmjopen.bmj.com/site/about/guidelines.xhtml

Table 2. Summary Topic Guides for professional, managerial and support staff, and patient interviews

**Health professionals, managerial and support staff**	**Role in the AKI prevention project**<br>• Current role<br>• How supported patients to prevent AKI before the project?<br>• Preparation for role in sick day guidance/AKI project<br>• Specific training/education<br>• Additional needs for training/education in the area of AKI prevention	**Views of the AKI prevention project**<br>• Who offered sick day rules/other AKI interventions to? (types of patient)<br>• How did you engage with patients<br>• What works well and why? (enablers)<br>• What does not work well and why? (barriers)<br>• Views of its impact on patients<br>• Views of the impact on your work, and the rest of the healthcare team	**Integration with health care**<br>• How do sick day rules/other AKI initiatives, fit/link with other support for AKI prevention?<br>• Fit with long term conditions management and other health needs and services?<br>• How do they fit/link with hospital care/social/voluntary sector?<br>• Contact/interaction with the rest of the primary health care team, secondary care team(s) around sick day guidance/AKI more generally?<br>• Which health care professionals are best placed to provide AKI prevention support?
**Patients**	**Context/history**<br>• Length of time of condition/taking medicines<br>• Perceptions of health and illness in everyday life<br>• Management of medicines and/or acute episodes of illness before the project (whether used a sick day guidance before/blister packs)<br>• Difficulties experienced around managing medicines and any needs?	**The sick day guidance/other kidney health interventions**<br>• How they found out about the service?<br>• Whether used the card or not?<br>• What do they find useful or like about it?<br>• What do they not find useful or dislike about it?<br>• Do they feel it has helped them? If so, how?<br>• Could it be improved? If so, how?<br>• Which healthcare practitioners could/should provide the cards? (where and when)<br>• Who are sick day cards/other AKI interventions suitable for?	**Coordination of care**<br>• Who is involved in their care?<br>• How/where does the sick day guidance/other support provided as part of the project, fit with other services or care received or other self-care undertaken?

For peer review only - http://bmjopen.bmj.com/site/about/guidelines.xhtml

Data Analysis

A-M M developed a thematic analysis framework using the evaluation objectives and the four core constructs of Normalisation Process Theory (NPT) to understand implementation.[1516] NPT is concerned with social action rather than attitudes, its four core constructs are coherence (sense-making), cognitive participation (relational work), collective action (operational work) and reflexive monitoring (appraisal). [1516] The NPT constructs provided a pragmatic structure to consider different types of work surrounding the implementation of sick day guidance cards. Furthermore, it provided a sensitising framework to explore the relationships between different types of work being undertaken. [18]The questions asked of the health practitioner interview data included:

- how do they make sense of implementing the sick day card initiative? (coherence)
- what work have they done to implement the initiative? (operational work)
- how is the initiative being communicated or enacted by local others? (relational work)
- what judgments have been made about the initiative? (appraisal)

The questions we asked of the patient participant data included:

- how does the participant make sense of health and illness? (coherence)
- what was the context of the participant receiving a card and guidance?
- how did they make sense of the card and implement the guidance in their day to day lives? (coherence, operational, relational work)
- how did they value the intervention? (appraisal).

As the interviews were completed and transcribed, data from each account was grouped according to role, which resulted in six data sets: GP; practice nurse and health care

For peer review only - http://bmjopen.bmj.com/site/about/guidelines.xhtml

assistant; administration; community pharmacist; practice pharmacist; and patient-participant. Thematic analysis using the transcripts, the audio recordings and the field notes was carried out by A-M M and TB. Each interview within a role group was analysed, and the findings were compared with those within the same group. Variations and similarities in context, sense-making, implementation and appraisal of the card were noted, explored and compared with the findings within and between role groups to enhance broader understanding. [19] Key themes and tensions underpinning implementation emerged through comparative, contextual analysis of individual and collective working practises underpinning introduction of sick day guidance cards.

Results

A version of the findings of this paper is included in a wider report that has been provided to the funding organisation.[20] Acute Kidney Injury (AKI) was viewed as a new phenomenon and the implementation of sick day guidance cards entailed a new set of working practises. Analysis indicated that AKI prevention guidance was not necessarily a straightforward concept to understand, or to communicate. Health practitioners thought the cards required some knowledge of illness symptoms and medicines, and that patients had to decide how severe the symptoms were before acting, or re-starting their medication. One practice pharmacist stated '*…patients don't understand what fever is…they think that if they've got a headache it's fever…we're trying to explain and they don't understand, or they say well, if I had a bout of diarrhoea do I stop the medication…it's severe. Well, what is severe, you know? Obviously it's very subjective…*' (SKHIP13PP).

For peer review only - http://bmjopen.bmj.com/site/about/guidelines.xhtml

Comparative analysis highlighted a tension between the need to achieve reach to the populations deemed at risk (i.e. those taking medicines specified on the card) and at the same time ensure comprehension concerning use of the guidance. There was evidence that this tension influenced the implementation of the sick day guidance intervention. The following sections describe the different approaches employed.

Administration of the sick day guidance card in conjunction with face-to-face communication

A common theme was health professionals and patients valuing the need to explain the guidance in person. One patient reflected *'I don't think that it should be just put on a counter… I don't think, number one, they'll read it, number two, they'll digest what's on it, or number three, they'll apply it to themselves'* (SKHIP22PA). A practice nurse thought dialogue was also important to reduce miscommunication, avoid patient confusion and additional GP workload.

'I always explain …There's no point giving someone a card if they don't understand what it's for…my grandma wouldn't understand that. She'd probably misinterpret that and…stop taking everything' (SKHIP25PN).

Analysis of health practitioner and patient accounts revealed that patients responded to the guidance in a variety of ways, not always as intended. One patient participant used the terms sickness and illness interchangeably and spoke of different classifications of illness. She asked which type the guidance card was referring to, to be confident of following the instructions properly.

For peer review only - http://bmjopen.bmj.com/site/about/guidelines.xhtml

‘*What do you define as illness…? Well, I suppose I don't know… I've got arthritis, that's not an illness it's just a thing of life when you get older... I've had spinal surgery, but they're not illnesses*…’ (SKHIP22PA).

Two health practitioners reported instances of patients with medication associated diarrhoea stopping their tablets since receiving a card. This unintended consequence of the initiative, lead to those patients being prescribed alternative medication to alleviate the side effect. A couple of patient participant accounts revealed a lack of willingness to follow the guidance as it had not been implemented by their hospital specialist, whose opinion they trusted, and they did not want to make their condition worse. *‘I’d rather feel sick than have a problem with the high blood pressure…’* (SKHIP31PA).

The concept of temporary cessation of medicines required careful consideration, for example when to stop, restart and what dosage to reinstate. ‘*We don’t have enough data or…best practice… if you stop the metformin or whatever medication how long do you stop it for…? Then after a week are you going to restart them again on the ten milligram or are you going to start them on the 1.5, the 2.5…?*’ (SKHIP14GP).

Although valued by the health practitioners interviewed, implementation of sick day guidance initiative demanded extra work. In general practice, this was deemed less problematic when it fitted into existing long-term condition review appointments, particularly with practice nurses or health care assistants. In community pharmacies, implementation sat more readily within face-to-face medication review appointments or

For peer review only - http://bmjopen.bmj.com/site/about/guidelines.xhtml

opportunistic over-the-counter interactions, including the purchase of non-steroidal anti-inflammatory drugs (NSAIDS, such as ibuprofen). One community pharmacist used the purchase of anti-diarrhoeal or sickness medications as an opportunity to administer AKI guidance.

'*…when people have been coming in to buy stuff for sickness or diarrhoea… If it turns out that they're on one of the medications that's on the card, then we'll give them a card then as well and explain about it*' (SKHIP5CP).

There were limits to the implementation of sick day guidance in patient populations deemed at increased risk of AKI. Concerns were expressed across the health professionals interviewed that the cards and temporary cessation of medications were not suitable for patients with cognitive impairments such as Alzheimer's disease, reduced literacy in English, those with advanced learning difficulties or visual impairments, or for elderly housebound patients taking multiple medicines. One community pharmacist commented on the difficulties facing patients and carers using dosette box (blister pack) systems.

'*they* (patients) *might have four or five tiny little white ones, and then if they're elderly or they can't see the markings, they don't know what tablet they should be stopping…. if it was a family member looking out for it, that would be I guess possible, but a lot of the carers are not allowed to alter any medication'* (SKHIP7CP).

For peer review only - http://bmjopen.bmj.com/site/about/guidelines.xhtml

Administration of sick day guidance cards to patients in conjunction with telephone consultations

Phase Two of the project entailed Practice Pharmacists supporting the implementation of the sick day guidance cards in general practices (see Table 1). All of the four CCG employed pharmacists valued and engaged with the project. However, they outlined difficulties fitting the implementation in with their pre-existing workload. There were more patients to work with than anticipated, and the searches, writing to patients, communicating with them and feeding the results back to GPs took longer to complete than the pharmacists described having time for.

To implement the project in this context, a decision was made to have telephone conversations with patients rather than face-to-face interactions. However, this created additional challenges. The phone calls took as long as the face-to-face encounters as the pharmacists expressed a professional need to do things '*properly*'. They reported patients not always being happy to talk with a perceived stranger on the phone about their health. Patient understanding was harder to assess and patients did not necessarily agree to enact the guidance if they became ill. Unlike the face-to-face GP and practice nurse consultations, patients on the other end of the phone had no prior trusting relationship with the practice pharmacist. One pharmacist tried to mitigate some of these issues by talking with a GP in advance of phoning:

'…I'm not going to just pick up the phone and ring this patient now, I'm going to ask the GP what he thinks… for the slightly elderly- some patients, perhaps mental health issues….They obviously know their patients much better than I do so I always take their advice' (SKHIP11PP).

For peer review only - http://bmjopen.bmj.com/site/about/guidelines.xhtml

The community pharmacists also spoke of the difficulties of assessing patient comprehension in this way. *'I've had to phone patients …if you've got a query or the prescription will be changed or we'll want to question something …sometimes they're on the ball, they completely know, and sometimes they're just so confused'* (SKHIP7CP).

Sick day guidance cards being administered without verbal or written communication

Instructions administered to health practitioners (Figure 3) stressed the need for dialogue with patients to check understanding. However, accounts indicated that this did not always occur. Reasons included other work demands during a practice based consultation, limited time for dialogue, forgetting to discuss it, and some lack of confidence about what to say, partly because of the limited evidence base and so as not to confuse patients, especially those where less fluent in English '*we have quite a lot of different ethnicities here…they've got limited English I think they're not quite sure and it takes quite a while explaining …about what medicines to stop, when to stop it, when to restart it…*' (SKHIP10PN).

Though the community pharmacists were willing to talk with patients about the guidance cards, time shortages and other work demands impinged on implementation. One community pharmacist stated '*Half the time it's remembering to do it because you're thinking about that many different things*' (SKHIP5CP). In addition, they did not always have face-to-face contact with patients '*we've got like 900 of our own patients and we just make the packs and then send them out and delivery, so we don't actually have that much patient contact*' (SKHIP7CP).

For peer review only - http://bmjopen.bmj.com/site/about/guidelines.xhtml

Some health practitioners felt that the cards were self-explanatory. One practice nurse said '*vomiting is vomiting and diarrhoea is diarrhoea*' (SKHIP25PN). However, others did not agree. One GP thought it was really important to provide patients with written material to aid understanding and compliance '*with certain other sort of medicine regimes, we ask them to stop temporarily if there's a drug interaction and patients are okay with that, as long as you give them sort of written instructions and they know exactly why they're stopping. A lot of it is to do with the understanding. They don't like stopping things if they don't understand why…*' (SKHIP20GP).

A couple of patient accounts referred to finding cards in public information areas of medical practices and community pharmacies. One patient who found a card in this manner wanted to share the sick day guidance message '*…I went into the pharmacy last week, they were on the counter…I picked one up and brought it home …I think it's such a good idea that I've given one to my sister*' (SKHIP22PA).

Communication of AKI risk, but limited use of a sick day guidance card

One GP worked exclusively with patients in care homes across the CCG, which included patients who were diagnosed with cognition limiting conditions such as dementia. Though the guidance messages were deemed pertinent to these groups of patients more vulnerable to AKI, their use was limited due to a potential lack of understanding. *'So we have the card. We didn't use it a lot…We used it to give to the carers. I used it to give to a few of the patients that have capacity'* (SKHIP14GP).

For peer review only - http://bmjopen.bmj.com/site/about/guidelines.xhtml

The need for appropriate training for carers, nursing staff and associated social workers was raised, beyond the level of the sick day guidance card. Specifically there was felt to be an on-going need for health practitioners to highlight the importance of fluid management in conjunction with medicines management. '*…they* (dementia patients) *ended up not eating or drinking, worsening of the renal function and become unwell and they end up in hospital…' So it's working with the carer as well to understand…. It's serious things that they might die from, not being hydrated'* (SKHIP14GP).

Discussion

Principal Findings

Implementation of sick day guidance cards to prevent community based AKI entailed a new set of working practises. The temporary cessation of medicines during episodes of acute illness was not necessarily a straightforward concept to understand or communicate. Comparative analysis of participants' accounts highlighted a tension between ensuring reach in administration of the cards to at risk populations whilst being confident to ensure patient understanding of their purpose and use.

Strengths and Weaknesses of this study

Unlike an earlier study[21] a key strength of this evaluation was to conduct an in-depth exploration of systematic roll out across a single healthcare setting. The study was hypothesis generating and use of Normalisation Process Theory provided a sensitising framework for data collection and analysis.[15-17] Recognising that all theories have the potential to structure and constrain analysis, NPT was chosen as it ensured that a range of

For peer review only - http://bmjopen.bmj.com/site/about/guidelines.xhtml

individual and collective working practises were considered during analysis.[1516] Methods to enhance the trustworthiness of the findings, including their transferability, entailed exploring types of work undertaken in both general practices and community pharmacies as well as their use by a range of health professionals in these different settings. [22]

The study entailed comparative analysis of both patient and professional accounts in order to explore their use in clinical interactions as well as in everyday life. Thematic analysis has illuminated a key tension between achieving reach whilst ensuring comprehension of the card and its instructions. However, a larger sample size might have resulted in the identification of additional themes that may have had an impact on this theoretical framework. Further research is required to enhance patient understanding and use. Professional accounts allowed descriptions of experiences of use by patients, though difficulties were encountered recruiting patient participants who had experiences of having used a sick day guidance card at times of acute illness. It is important to acknowledge that only five patients were interviewed in spite of extensive recruitment efforts. It is not possible to determine how many patients received information packs as we did not ask practices to keep a record, to reduce work load. Health professionals did not always pass on the evaluation recruitment packs to patients, and the patients we interviewed had not used the cards to date; which could help to explain limited patient involvement. Workload pressures were cited as reasons for health professionals declining to participate in the evaluation.

During the course of the interviews, health practitioners were asked about patient sense-making, use and appraisal of the guidance cards. In light of limited patient involvement these accounts became more important. We acknowledge that they are third order

For peer review only - http://bmjopen.bmj.com/site/about/guidelines.xhtml

interpretations; our interpretations of what health practitioners reported about patients' sense-making, appraisal and use of the cards. However, the comparative approach taken has facilitated understanding of the pluralistic journeys of the cards and their intended and unintended messages and trajectories from card giver to patient across the 29 interviews. Future studies may benefit from sampling patients who have been coded in general practice as having been provided sick day guidance (i.e. Read Code 8OAG. 'Provision of information about Acute Kidney Injury'[23]) and also who have been coded with an episode of acute illness (e.g. gastroenteritis, acute respiratory infection). In doing so, this this would enable purposeful sampling according to medical history including evidence of multi-morbidity. As stated in the CCG report, 106,000 cards (see Table 1) were distributed across general practices and community pharmacies within the time frame of the project. [20] However, community pharmacists were not required to record administration to patients and inaccuracies in coding in general practice limited the potential for a robust quantitative analysis. Future study design would benefit from greater alignment between quantitative and qualitative elements of an evaluation. [20]

Comparison with other studies

In terms of professional responsibility, there are recognised boundaries to the role of general practitioners in supporting self-management.[24] The findings of this study resonate and build on the results of previous research, which highlighted issues around the consistency of clinical message, and the additional work required to reduce the risk of harm from AKI using medicines management interventions.[2125] The intervention was conducted at a time when concern was raised that UK general practice workload may be at 'saturation point.'[26] Results suggested that this influenced engagement with the CCG led initiative.

For peer review only - http://bmjopen.bmj.com/site/about/guidelines.xhtml

Though currently available through the Scottish Patient Safety Programme,[6] the findings from this qualitative study resonate with recently published literature, which highlights a need for a more robust evidence base surrounding both the implementation and effectiveness of sick day guidance cards.[27-29] A recent systematic review showed that 'there is no evidence of the impact of drug cessation interventions on AKI incidence during inter-current illness in primary or secondary care.'[2729] In terms of implementation, studies evaluating AKI interventions in secondary care indicate that establishing clinician approval is critical with a need for intervention design to take into account 'how technologies, people and organisations dynamically interact' in order for AKI interventions to become integrated into routine clinical practice. [3031]Interventions that disrupt workflow 'may not be sustainable even if there has been a positive impact on care.'[30]

Results from a population-based cohort study indicate that patient co-morbidities including chronic kidney disease are much more strongly associated with AKI and that treatment with either an ACE Inhibitor or an ARB is only associated with a small increase in AKI risk.[28]That is, younger patients with limited comorbidity (e.g. on ACEI for treatment of hypertension) have a low absolute risk of AKI, whilst patients living with multi-morbidity in whom there may be professional concerns about ensuring effective risk communication, have a much higher risk of AKI. [28]

Implications for clinicians, policy makers and future research

In the UK, NICE recommends raising awareness of AKI in higher risk population groups with specific reference to patients who: have existing CKD; have had a previous episode of illness

For peer review only - http://bmjopen.bmj.com/site/about/guidelines.xhtml

complicated by AKI; and/or have neurological or cognitive impairment and who may be reliant on carers for support with fluid intake during an acute illness (e.g. those with cognitive impairment).[32] This may help address a knowledge gap in patient and public understanding of the importance in the maintenance of kidney health. A survey conducted in 2014 on behalf of NHS England indicated that 'about half of the population in Great Britain don't think their kidneys make urine' and 'only an eighth (12%) of interviewees thought their kidneys had a role in processing medicines.'[33] However, the findings from this study suggest an evidence base is urgently warranted to determine how best to resource effective self-management support for higher risk patient populations. Targeting patients who have had an episode of illness complicated by AKI may be particularly important. As a marker of vulnerability, data from a Welsh study showed that around 50% of their patient population died within 14 months; the study also revealed high rates of hospital readmission.[34] Of the 733 patients discharged following a hospital admission complicated by AKI, there were 498 rehospitalisation events in a six month period.[34]

The NHS England Urgent and Emergency Care Review also emphasised the need for better support for people to self-care.[35] Our analysis in conjunction with the research by Mansfield et al (2016),[28] suggest sick day guidance cards alone, that focus solely on temporary cessation of medicines, are unlikely to be sufficient to reduce the harm associated with AKI. The CCG chose to implement the Scottish (NHS Highland) Medicine Sick Day Rules card without significant modification of content or format.[6] However, the current intervention may need modifying, to make it suitable for use with various populations, such as provision in languages other than English. For example, recognising the risks of the 'triple whammy' combination of NSAIDS prescribed in conjunction with diuretics and renin–angiotensin system inhibitors (i.e. ACE inhibitors and ARBs), is there potential for misunderstanding if

For peer review only - http://bmjopen.bmj.com/site/about/guidelines.xhtml

NSAIDS are included in a sick day guidance card administered to patients with heart failure?[36] Both usability testing as well as experience based co-design are methodological approaches that may optimise the development of an intervention that takes into account patient and carer experience.[37] The findings suggest other strategies may need to be resourced to prevent AKI in people with complex health and social care needs such as those living with dementia. A key issue raised was to provide better education and support for carers (both professional and informal). The Royal College of General Practitioners has provided guidance on the development of 'carer friendly' practises and the establishment of Patient Participation Groups, may be a mechanism to resource and integrate support for carers into the organisation of acute care.[3839]

Conclusions

The findings from this qualitative evaluation suggest that there are boundaries to the implementation of sick day guidance cards to prevent Acute Kidney Injury in primary care. A common theme was the need to ensure patient understanding of their purpose and use. Communicating the concept of temporary cessation of medicines was a particular challenge and limited their administration to patient populations at higher risk of AKI, particularly those with less capacity to self-manage. The analysis suggests that sick day guidance cards that focus solely on medicines management may be of limited benefit without either adequate resourcing, or if delivered as a standalone intervention. Development and evaluation of a primary care intervention encompassing a range of initiatives to tackle the harm associated with AKI is warranted.

For peer review only - http://bmjopen.bmj.com/site/about/guidelines.xhtml

Figure Legends

Figures 1 (front) and 2 (back) Sick day guidance card used during this project

Image of front of sick day card Figure1, and back of sick day card Figure 2

The NHS Highland sick day rules card was reproduced with new logos [6-8] (text to be placed under images)

Figure 3 Guidance provided to health practitioners (shortened form)

Declarations

Ethics approval and consent to participate

Ethical approval was gained from Leeds West Research Ethics Committee (REC Reference Number: 15/YH/0174). Informed consent was gained from all participants prior to interview.

Consent for publication

Not applicable.

Availability of data and material

The data has been stored securely with password protected files to ensure confidentiality, in keeping with the research protocol and good data management guidelines. It will not be shared.

Competing interests

The authors declare that they have no competing interests.

For peer review only - http://bmjopen.bmj.com/site/about/guidelines.xhtml

Funding

This project was funded by the National Institute for Health Research Collaboration for Leadership in Applied Health Research and Care (NIHR CLAHRC) Greater Manchester and NHS Salford CCG. The NIHR CLAHRC Greater Manchester is a partnership between providers and commissioners from the NHS, industry and the third sector, as well as clinical and research staff from the University of Manchester. The views expressed in this article are those of the authors and not necessarily those of the NHS, NIHR or the Department of Health".

Authors Contributions

TB, RE, SS, SM and SH conceived and designed the study, A-MM and RE collected the data, TB and A-MM analysed the data. A-M M, RE and TB all have extensive experience of designing, conducting and analysing qualitative health research at doctoral and post-doctoral level. All authors contributed to writing the manuscript and have read and approved the final manuscript.

Acknowledgements

The authors would like to thank the project steering group for their input and guidance throughout the study.

Authors' information (optional)

TB is a member of NHS England's Think Kidneys Programme Board.

For peer review only - http://bmjopen.bmj.com/site/about/guidelines.xhtml

For peer review only

For peer review only - http://bmjopen.bmj.com/site/about/guidelines.xhtml

References

1. Mehta RL, Cerdá J, Burdmann EA, et al. International Society of Nephrology's 0by25 initiative for acute kidney injury (zero preventable deaths by 2025): a human rights case for nephrology. *The Lancet*;385(9987):2616-43.

2. Kidney Disease Improving Global Outcomes (KDIGO) Acute Kidney Injury Work Group. KDIGO Clinical Practice Guideline for Acute Kidney Injury. *Kidney International Supplement* 2012;2(1):1–138.

3. NHS England, UK Renal Registry, Think Kidneys. Acute Kidney Injury Best Practice Guidance: Responding to AKI Warning Stage Test Results for Adults in Primary Care: Think Kidneys, 2016.

4. National Institute for Health and Care Excellence. Acute Kidney Injury: prevention, detection and management (CG169): NICE, 2013.

5. NHS England, UK Renal Registry, Think Kidneys. Acute Kidney Injury Warning Alert Best Practice document: Think Kidneys, 2014.

6. NHS Scotland, Health Improvement Scotland, Scottish Patient Safety Programme. Medicine Sick Day Rules Cards [internet]. NHS Scotland, Health Improvement Scotland; 2016 Availablefrom:http://www.scottishpatientsafetyprogramme.scot.nhs.uk/programmes/primary-care/medicine-sick-day-rules-card [accessed 4 April, 2017].

7. Kerr M, Bedford M, Matthews B, et al. The economic impact of acute kidney injury in England. *Nephrology Dialysis Transplantation* 2014;29(7):1362-68.

8. Morrison C, Wilson, M. Medicine Sick Day Rules Cards Intermin Evaluation. [internet] NHS Highland. July 2014 Available from http://www.knowledge.scot.nhs.uk/media/CLT/ResourceUploads/4055542/NHSH%20interim%20evaluation%20medicine%20sick%20day%20rules.pdf [accessed 7 January 2015].

9. Feehally J, Gilmore I, Barasi S, et al. RCPE UK consensus conference statement: management of acute kidney injury: the role of fluids, e-alerts and biomarkers. *J R Coll Physicians Edinb* 2013;43 doi: 10.4997/jrcpe.2013.109.

For peer review only - http://bmjopen.bmj.com/site/about/guidelines.xhtml

10. Scottish Government. NHS Scotland Polypharmacy guidance [internet]. March 2015. Available from: http://www.sehd.scot.nhs.uk/publications/DC20150415polypharmacy.pdf [accessed 2 April 2015].

11. Morrison C, Wilson M. Medicine Sick Day Rules cards: a safe and effective tool to improve medicines safety in NHS Highland [internet]. *International Journal of Pharmacy* Practice 2015;23;S2. DOI: 10.1111/ijpp.12213. Available fromhttp://onlinelibrary.wiley.com/doi/10.1111/ijpp.12213/pdf.

12. NHS England, UK Renal Registry. [internet] Think Kidneys Available from http://www.thinkkidneys.nhs.uk/ accessed 7 Apr 2015.

13. NHS England, UK Renal Registry, Think Kidneys. "Sick day" guidance in patients at risk of Acute Kidney Injury: an Interim Position Statement from the Think Kidneys Board: Think Kidneys, 2015.

14. Hoffmann TC, Glasziou PP, Boutron I, et al. Better reporting of interventions: template for intervention description and replication (TIDieR) checklist and guide. *BMJ* 2014;348 doi: 10.1136/bmj.g1687.

15. May C, Mair F, Finch T, et al. Development of a theory of implementation and integration: normalisation process theory. *Implement Sci* 2009;4.

16. Murray E, Treweek S, Pope C, et al. Normalisation process theory: a framework for developing, evaluating and implementing complex interventions. *BMC Med* 2010;8:63.

17. normalizationprocess.org [internet]. Normalization Process Theory On-line Users' Manual, Toolkit and NoMAD instrument; 201.5 Available from http://www.normalizationprocess.org [accessed 10 October 2015].

18. Jung JY, Blakeman T, Hegarty J, Humphreys J, Harvey G. Understanding the implementation of interventions to improve the management of chronic kidney disease in primary care: a rapid realist review. *Implementation Science* 2016;11:47. DOI: 10.1186/s13012-016-0413-7.

19. Silverman S. Interpreting Qualitative Data: Methods for Analysing Talk, Text and Interaction. London: Thousand Oaks 2001.

20. NIHR Collaboration for Leadership in Applied Health Research and Care Greater Manchester. Salford Clinical Commissioning Group Sick Day Guidance Project: Final Report. Manchester: NIHR CLAHRC GM, 2016.

For peer review only - http://bmjopen.bmj.com/site/about/guidelines.xhtml

21. Morris RK AD, Phipps D, Bower P et al. Preventing Acute Kidney Injury: A qualitative study exploring 'sick day rules' implementation in primary care. *BMC Family Practice* 2016;FAMP-D-15-00010R3 (Accepted for Publication 12 July 2016)

22. Lincoln Y, Guba EG. Naturalistic Inquiry. London: Sage; 1985.

23. NHS North West Commissiong Support Unit. Salford Standards Read Code Dictionary. Specification for Read V2 coding for: Salford Clinical Commissioning Group: NHS North West Commissioning Support Unit, 2016.

24. Blakeman T, Macdonald W, Bower P, et al. A qualitative study of GPs' attitudes to self-management of chronic disease. *Br J Gen Pract* 2006;56(527):407-14.

25. Phipps DL, Morris RL, Blakeman T, et al. What is involved in medicines management across care boundaries? A qualitative study of healthcare practitioners' experiences in the case of acute kidney injury. *BMJ Open* 2017;7(1) doi: 10.1136/bmjopen-2016-011765

26. Hobbs FDR, Bankhead C, Mukhtar T, et al. Clinical workload in UK primary care: a retrospective analysis of 100 million consultations in England, 2007-14. *The Lancet* 2016; 287 (10035): 2323-2330.

27. Morden A, Horwood J, Whiting P, et al. The risks and benefits of patients temporarily discontinuing medications in the event of an intercurrent illness: a systematic review protocol. *Systematic Reviews* 2015;4(1):1-6. doi: 10.1186/s13643-015-0135-y

28. Mansfield KE, Nitsch D, Smeeth L, et al. Prescription of renin–angiotensin system blockers and risk of acute kidney injury: a population-based cohort study. *BMJ Open* 2016;6(12) doi: 10.1136/bmjopen-2016-012690

29. Whiting P MA, Tomlinson LA, Caskey F, et al. What are the risks and benefits of temporarily discontinuing medications to prevent acute kidney injury? A Systematic Review and meta-analysis. *BMJOpen* 2017;bmjopen-2016-012674.R1

30. Kanagasundaram NS, Bevan MT, Sims AJ, Heed A, Price DA, Sheerin, NS. Computerized clinical decision support for the early recognition and management of acute kidney injury: a qualitative evaluation of end-user experience. *Clin Kidney J* (2016) 9 (1): 57-62. DOI: https://doi.org/10.1093/ckj/sfv130.

For peer review only - http://bmjopen.bmj.com/site/about/guidelines.xhtml

31. Oh J, Bia JR, Ubaid-Ullah M, Testani JM, Wilson FP. Provider acceptance of an automated electronic alert for acute kidney injury. *Clin Kidney J* (2016) 9 (4): 567-571. DOI: https://doi.org/10.1093/ckj/sfw054.

32. National Institue for Health and Care Excellence. Acute kidney injury Quality standardnice.org.uk/guidance/qs76: NICE, 2014.

33. NHS England, UK Renal Registry, Think Kidneys. Understanding what the public know about their kidneys and what they do. Findings from Ipsos MORI survey – July 2014: Think Kidneys, 2014.

34.Wonnacott A, Meran S, Amphlett B, Talabani B, Phillips A. Epidemiology and outcomes in community-acquired versus hospital-acquired AKI. *Clin J Am Soc Nephrol*. 2014 Jun 6;9(6):1007-14. doi: 10.2215/CJN.07920713.

35. NHS England. High quality care for all, now and for future generations: Transforming urgent and emergency care services in England, Urgent and Emergency Care Review. Leeds: Urgent and Emergency Care Review Team, NHS England, 2013.

36. Dreischulte T, Morales DR, Bell S, Guthrie B. Combined use of nonsteroidal anti-inflammatory drugs with diuretics and/or renin-angiotensin system inhibitors in the community increases the risk of acute kidney injury. *Kidney Int*. 2015 Aug;88(2):396-403. doi: 10.1038/ki.2015.101

37. Locock L, Robert G, Boaz A, et al. Testing accelerated experience-based co-design: a qualitative study of using a national archive of patient experience narrative interviews to promote rapid patient-centred service improvement. Southampton (UK): NIHR Journals Library; 2014 Feb. (*Health Services and Delivery Research*, No. 2.4.) Available from: https://www.ncbi.nlm.nih.gov/books/NBK259580/ doi: 10.3310/hsdr02040

38. Royal College of General Practitioners. RCGP Supporting Carers in General Practice: Summary report on GP practice journeys towards improved carer identification and support. London: Royal College of General Practitioners, 2013.

39. NHS Employers. 2015/16 General Medical Services (GMS) contract: Guidance for GMS contract 2015/16: NHS Employers, 2015.

For peer review only - http://bmjopen.bmj.com/site/about/guidelines.xhtml

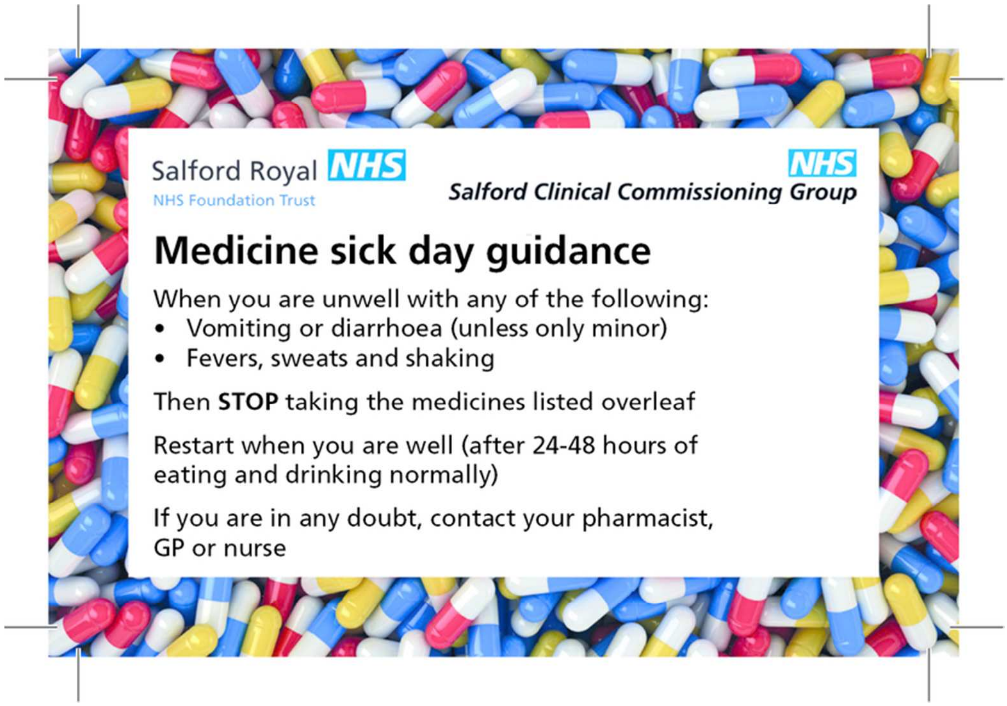

Figure 1 (front) Sick day guidance card used during this project 6-8

69x48mm (300 x 300 DPI)

For peer review only

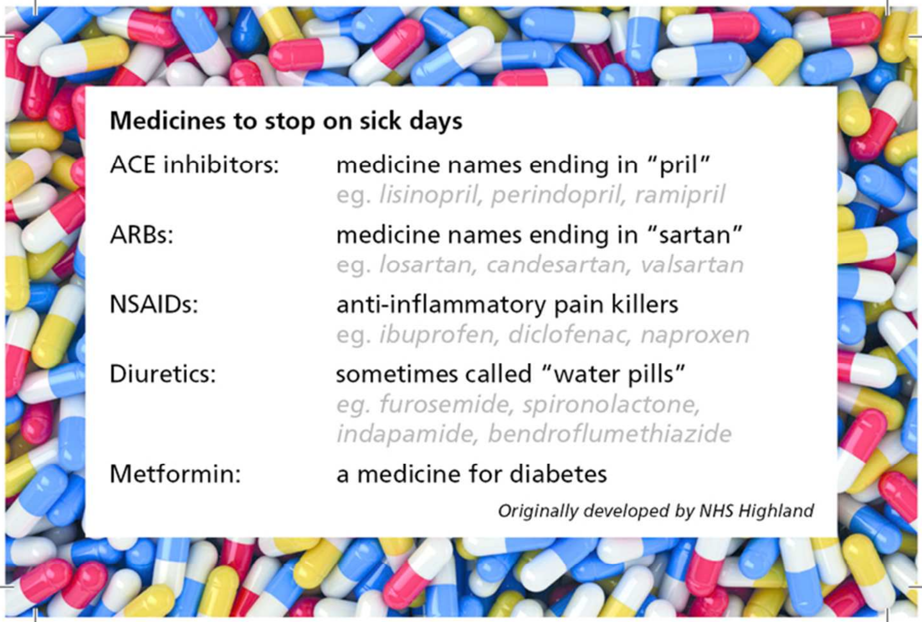

Figure 2 (back) Sick day guidance card used during this project

69x49mm (300 x 300 DPI)

MEDICINES AND DEHYDRATION: SICK DAY GUIDANCE

Offer the following information at the time of giving the card

- Some medicines shouldn't be taken when you have an illness that makes you dehydrated. This is because they can either increase the risk of dehydration or because dehydration can lead to potentially serious side effects of the medicine.
- The medicine you are taking that falls into this category is [tell patient which medicine].
- Illnesses that can cause dehydration are vomiting, diarrhoea and fever.
- This advice does not apply to minor sickness or diarrhoea, which means a single episode.
- If your medicines are in a blister pack you must take it to the chemists so the chemist can show you which ones you need to stop.
- If you have heart failure you may stop these medicines for a maximum of 48 hours but after that you need to contact your GP or heart failure team for further advice.

The list of medicines on the card is not exhaustive but they are highlighted because:

- diuretics can cause dehydration or make dehydration more likely in an ill patient;
- ACE inhibitors, angiotensin II receptor blockers and NSAIDs may impair kidney function in a dehydrated patient, which could lead to kidney failure;
- metformin dehydration increases the risk of lactic acidosis, a serious and potentially life-threatening side effect of metformin.

Figure 3 Guidance provided to health practitioners (shortened form)

Understanding the implementation of 'sick day guidance' to prevent Acute Kidney Injury across a primary care setting in England: a qualitative evaluation

COREQ Table

Guide question		Response	Manuscript page number
1	Interviewer/facilitator	The interviews were conducted by AM-M and RE.	11
2	Credentials	AM-M Ba, Ma, PhD<br>RE Ba, Ma, PhD<br>SH BSc, PhD<br>SM MBChB<br>SS MBChB MRCP(UK) PhD<br>TB MBChB; PhD	1-2
3	Occupation	A-M M Post-doctoral research associate, Collaboration for Leadership in Applied Health Research and Care Greater Manchester) (CLAHRC GM),<br>RE Research Fellow, CLAHRC GM,<br>SH Kidney Research Programme Manager, CLAHRC GM<br>SM Local clinical research speciality lead and GP, NHS Salford Clinical Commissioning Group<br>SS Senior renal consultant, Salford Royal NHS Foundation Trust<br>TB Clinical Senior Lecturer and GP, CLAHRC GM.	1-2
4	Gender	A-M M female<br>RE female<br>SH female<br>SM female<br>SS female<br>TB male	1-2
5	Experience and training	A-M M is a trained social anthropologist and experienced health researcher<br><br>RE is an experienced and trained qualitative researcher in health<br><br>TB is an experienced and trained qualitative health researcher<br><br>SH is an experienced post-doctoral health research manager<br><br>SS and SM are both health professionals with experience of designing kidney health research and interventions.	29<br><br>29<br><br>29<br><br>Tidier Table page 8, item 5 'Who'<br><br>Tidier Table page 8, item 1 'Why', item 5 'Who'.

For peer review only

Guide question		Response	Manuscript page number
6	Relationship established	The researchers conducting the interviews (A-M M, RE) did not know any of the interviewees prior to the research.	11
7	Participant knowledge of the researcher	Via information sheets and verbal clarification the participants knew that A-M M and RE were researchers evaluating the acute kidney injury (AKI) sick day guidance initiative.	11
8	Researcher characteristics	The researchers were working on a (CLAHRC) programme of research on kidney ill-health prevention in Greater Manchester.	Page 8, Tidier Table, item 1, 'Why'
9	Methodological orientation and theory	We used normalisation process theory (NPT) to inform the design in the interview topic guide and analysis, NPT aims to explore the contexts, sense-making, activity and participant appraisal of an implementation project.	Page 10, page 13 Table 2, and pages 14-15.
10	Sampling	Each pharmacy and GP practice in the locality was asked to take part in the evaluation and to help recruit patient participants via dissemination of information packs. We asked those we interviewed if they knew others who might want to take part.	10
11	Method of approach	Participants were contacted by email and post.	10 and 11
12	Sample size	29 participants.	10, 11
13	Non-participation	'Workload pressures were cited as reasons for health professionals declining to participate in the evaluation.'	23
14	Setting of data collection	Data were collected at the participants' place of work and over the phone.	11
15	Presence of non-participants	Not applicable.	
16	Description of sample	29 participants comprised of: seven GPs; five practice nurses; five community pharmacists; four practice based pharmacists; two managers (one medical practice manager and one community pharmacy manager); and a health care assistant.	10
17	Interview guide	See Table 2. Summary Topic Guides for professional, managerial and support staff, and patient interviews.	13
18	Repeat interviews	Not applicable.	
19	Audio/visual recording	Audio recorded, with consent from the participant, as explained in verbal and written information.	11
20	Field notes	Written after each interview and used to inform analysis.	11, 15
21	Duration	From 9 minutes to 66 minutes (median = 33 minutes).	11
22	Data saturation	Participants were recruited until saturation of key emergent theme. Practical consideration also determined end point in recruitment.	23
23	Transcripts returned	Participants were asked if they wanted a copy of the transcript, none did.	12
24	Number of data coders	2, data analysis was conducted by A-M M and TB.	14-15.
25	Description of the	Analysis was focused around NPT and the evaluation	14-15

For peer review only

Guide question		Response	Manuscript page number
	coding tree	objectives.	
26	Derivation of themes	Analysis was focused around the four constructs of NPT coherence (sense-making), cognitive participation (relational work), collective action (operational work) and reflexive monitoring (appraisal); the evaluation objectives and we allowed for serendipitous findings. Sense-making, activities and evaluation of card comprehension and use emerged through engagement with these constructs.	14-15, 15-22
27	Software	Ms Word (transcripts, and fieldnotes) and an audio recorder were utilised to record and help make sense of the data.	14-15
28	Participant checking	Participants were not asked to provide feedback on the findings.	
29	Quotations presented	Quotations are presented throughout the results section.	15-22
30	Data and findings consistent	The themes arising stem from the data and are consistent throughout.	15-22
31	Clarity of major themes	The major themes are presented under headings; and under the heading principal findings.	15-22, 22.
32	Clarity of minor themes	Minor themes are incorporated into the themes outlining how sick day guidance cards were implemented in routine practice.	15-22

For peer review only
